# Semiempirical Methods for Molecular Systems in Strong Magnetic Fields

**DOI:** 10.1021/acs.jctc.3c00671

**Published:** 2023-09-06

**Authors:** Chi Y. Cheng, Andrew M. Wibowo-Teale

**Affiliations:** † School of Chemistry, University of Nottingham, University Park, Nottingham NG7 2RD, U.K.; ‡ Hylleraas Centre for Quantum Molecular Sciences, Department of Chemistry, University of Oslo, P.O. Box 1033, Blindern, N-0315 Oslo, Norway

## Abstract

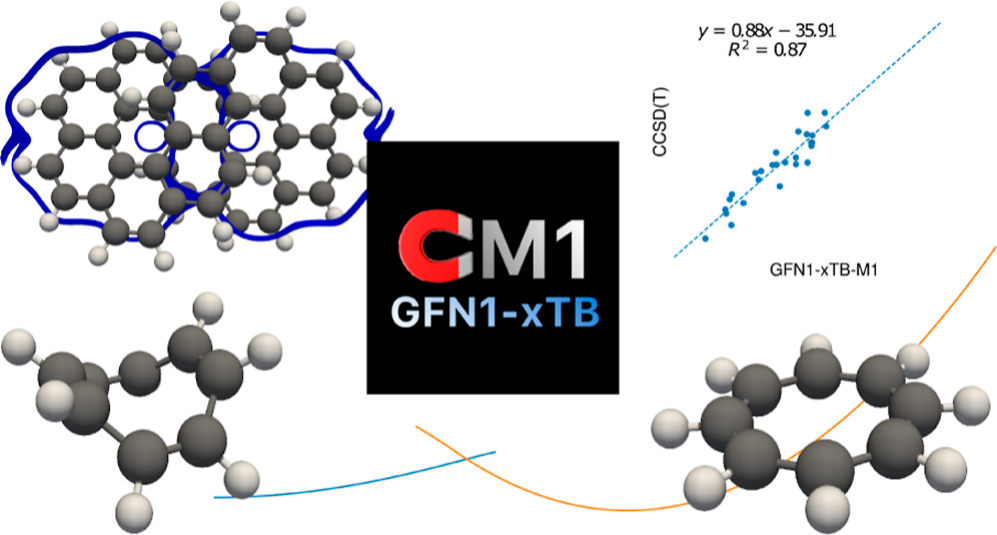

A general scheme is presented to extend semiempirical methods to include the effects of arbitrary strength magnetic fields, while maintaining computational efficiency. The approach utilizes three main modifications; a London atomic orbital (LAO) basis set is introduced, field-dependent kinetic energy corrections are added to the model Hamiltonian, and spin-Zeeman interaction energy terms are included. The approach is applied to the widely available density-functional tight-binding method GFN1-xTB. Considering the basis set requirements for the kinetic energy corrections in a magnetic field leads to two variants: a single-basis approach GFN1-xTB-M0 and a dual-basis approach GFN1-xTB-M1. The LAO basis in the latter includes the appropriate nodal structure for an accurate representation of the kinetic energy corrections. The variants are assessed by benchmarking magnetizabilities and nuclear magnetic resonance shielding constants calculated using weak magnetic fields. Remarkably, the GFN1-xTB-M1 approach also exhibits excellent performance for strong fields, || ≤ 0.2*B*_0_ (*B*_0_ = 2.3505 × 10^5^ T), recovering exotic features such as the para- to dia-magnetic transition in the BH molecule and the preferred electronic configuration, molecular conformation, and orientation of benzene. At stronger field strengths, || > 0.2*B*_0_, a degradation in the quality of the results is observed. The utility of GFN1-xTB-M1 is demonstrated by performing conformer searches in a range of field strengths for the cyclooctatetraene molecule, with GFN1-xTB-M1 capturing the transition from tub to planar conformations at high field, consistent with much more computationally demanding current-density functional theory calculations. Magnetically induced currents are also shown to be well described for the benzene and infinitene molecules, the latter demonstrating the flexibility and computational efficiency of the approach. The GFN1-xTB-M1 approach is a useful tool for the study of structure, conformation, and dynamics of large systems in magnetic fields at the semiempirical level as well as for preoptimization of molecular structure in ab initio calculations, enabling more efficient exploration of complex potential energy surfaces and reactivity in the presence of external fields.

## 1 Introduction

In recent years, a large number of approaches have been developed to allow for the study of atomic and molecular systems in strong magnetic fields.^1−23^ Several electronic structure packages have now been developed with the capabilities to allow a nonperturbative treatment of magnetic fields^24−27^ for a range of methods including Hartree–Fock (HF),^1,14^ configuration interaction,^3^ Møller–Plesset (MP),^28^ coupled-cluster (CC),^6^ equation of motion coupled-cluster,^10,11^ Green’s function-based GW,^22,29^ and current-density functional theories (CDFTs).^9,13,16,17,28,30^

In the presence of an external magnetic field, calculations are generally more expensive to carry out. This is primarily because the associated wave functions are generally no longer real but complex. This leads to a requirement for complex arithmetic in the numerical implementation of the electronic structure methods, as well as a loss of complex-conjugation symmetry resulting in an increased computational effort required for molecular integral and integral derivative evaluation over suitable complex basis functions, such as London atomic orbitals (LAOs).^31^ Furthermore, the presence of a magnetic field leads to a lowering of any point-group symmetry that may be exploited,^19,21,23,32^ particularly if general orientations of the molecular frame relative to the applied field are to be considered. To offset these challenges, significant progress has been made to exploit resolution-of-the-identity^16,18^ and Cholesky decomposition techniques^33,34^ in molecular integral evaluation.

The availability of techniques to accelerate integral evaluation over LAOs has made calculations on large systems much more tractable. However, despite these advances, calculations remain more demanding than their zero-field counterparts. Given that the effects of magnetic fields are expected to be observable at lower fields for larger systems, it is desirable to develop lower-cost approaches to increase the system size that is amenable to simulation. Recently, embedded fragment-based approaches were developed based on HF, CDFT, MP, and CC methods with LAO basis sets to address large, noncovalently bound, molecular clusters.^28^

In the present work, the development of semiempirical approaches for application to more general molecular systems in strong magnetic fields is explored. A general approach to yield semiempirical models, which can be directly compared with and assessed against ab initio methods using LAO basis sets, is developed. In particular, we focus on the popular density-functional tight-binding method GFN1-xTB,^35,36^ which we have adapted to include the effects of an arbitrary strength magnetic field in a nonperturbative manner. The history of similar approaches is extensive, and Section 2 briefly reviews the relevant background and theory. In Section 2.1, the basics of Hückel-London (HL) theory^31,37^ are introduced; in Section 2.2, we consider some modifications that can be made to the extended Hückel theory to treat strong magnetic fields. In Section 2.3, similar considerations are applied to the GFN1-xTB model, generating a flexible semiempirical approach suitable for applications to a wide variety of chemical systems in magnetic fields. Computational details are summarized in Section 3, and the applicability of the semiempirical models is assessed for magnetic properties in Section 4 with weak fields in Section 4.1 and strong fields in Section 4.2. Conclusions and directions for future work are presented in Section 5.

## 2 Background and Theory

### 2.1 Hückel–London Theory

The Hückel–London (HL) theory^31,37^ was one of the first approaches to include the interaction of a magnetic field in a semiempirical method. In the HL theory, the effect of a magnetic field is included by multiplying the resonance integral terms from Hückel theory by a field-dependent complex exponential, forming the following effective one-electron Hamiltonian for conjugated hydrocarbons
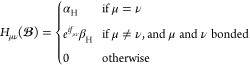
1where α_H_ and β_H_ are the Hückel Coulomb and resonance integral parameters and
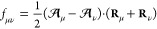
2where
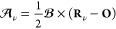
3is the vector potential associated with a uniform magnetic field, defined in the Coulomb gauge, evaluated at the atomic orbital (AO) center **R**_ν_. The magnetic field vector is , and the associated gauge-origin is **O**. London’s approach to extending Hückel theory to include magnetic fields has two particularly useful properties: the original semiempirical method is returned at zero-field (since  as ) and the total energies are independent of the gauge-origin since the field-dependent complex exponential is itself independent of the gauge-origin.

### 2.2 Extended Hückel Theory

A more recent example where the effect of a magnetic field has been included in a semiempirical method is the work by Hod et al.,^38,39^ who made modifications to the extended Hückel theory^40−45^ to include orbital paramagnetic and diamagnetic terms in the effective one-electron Hamiltonian, resulting in an approach called the magnetic extended Hückel theory (MEHT). Using MEHT, Hod et al.^38,39^ generated computational models of devices with molecular junctions featuring quantum corrals, carbon nanotubes, and molecular rings formed from polyaromatic hydrocarbons. The magnetoresistances of these devices were then investigated by applying an external magnetic field.^38,39^

Despite significant advances in the development of ab initio methods under a strong magnetic field, there is still a need for low-cost approaches, for example, to allow for the study of large systems. Although HL theory and MEHT could be used to model molecular systems under a strong magnetic field, there are some deficiencies in both methods. For example, the spin-Zeeman interaction terms are absent in both approaches and so open-shell systems (which can be particularly important in strong magnetic fields) are not modeled correctly. In addition, significant progress has been made in developing more accurate semiempirical models that are parameterized for a broad range of chemistry.^35,36,46−54^ In the present work, the aim is to develop a general set of modifications to extend any semiempirical method for applications in a magnetic field. We focus on the widely available modern density-functional tight-binding method GFN1-xTB^35,36^ as an example to create a robust alternative to the more computationally expensive ab initio methods for calculations under a magnetic field.

To develop a general set of modifications to include the effects of a magnetic field for semiempirical methods, we begin by considering the extended Hückel theory which has the energy expression
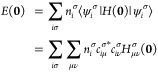
4where we have chosen to explicitly write the summation over the σ = {α, β} spin electrons. Here, ψ_*i*_^σ^ are the occupied valence molecular orbitals, *n*_*i*_^σ^ are the molecular orbital occupation numbers, and *c*_*i*μ_^σ*^ and *c*_*i*ν_^σ^ are the molecular orbital coefficients. Greek indices refer to AOs, ϕ_ν_, used to represent each molecular orbital as a linear combination, ψ_*i*_^σ^ = ∑_ν_*c*_*i*ν_^σ^ϕ_ν_. The Hamiltonian matrix elements in the AO basis for a system in the absence of a magnetic field, *H*_μν_^σ^(**0**), are given by
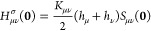
5where *K*_μν_ are a fitting parameters, *h*_μ_ and *h*_ν_ are valence state ionization potentials, and *S*_μν_(**0**) are the overlap integrals. The orbital coefficients are determined by solving the generalized eigenvalue problem
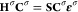
6

#### 2.2.1 Kinetic Energy Corrections and Gauge-Origin Independence

A naïve adjustment to include the effects of a magnetic field in the model Hamiltonian of eq 5 is to simply add a correction term consisting of the difference between the kinetic energy contributions in a magnetic field and those in the absence of a field, along with the spin-Zeeman interaction term
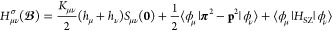
7Here, **p** is the momentum operator,  is the kinetic-momentum operator, and  is the spin-Zeeman operator which depends linearly on the magnetic field and the spin operator **S**. However, eq 7 is problematic for practical applications since in a finite basis set, the resulting energy can change with a change to the gauge-origin due to the integrals involving the **π**^2^ operators.

For ab initio methods, this problem can be overcome with LAOs which can be obtained from a set of AOs by multiplying them by a field-dependent complex exponential^31^
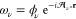
8where ω_ν_ are the LAOs. Unfortunately, changing all integrals to use LAOs so that we make the replacements ϕ_ν_ → ω_ν_ and  in eq 7 leads to a similar problem since there would then be a gauge-origin dependence in the integrals involving the **p**^2^ operators. This is clear if we write out the integral
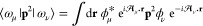
9since the evaluation of the partial derivatives in eq 9 will lead to terms that are linear and quadratic with respect to the gauge-origin.

One solution would be to add the field-dependent kinetic energy and spin-Zeeman interaction energy terms to eq 5, use LAOs for all integrals, and then subtract certain terms that appear in the field-dependent kinetic energy, specifically the integrals
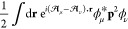
10which are the only terms in the field-dependent kinetic energy that do not disappear in the absence of a magnetic field and are also independent of the gauge-origin. However, this will lead to a Hamiltonian which is not Hermitian. Although the anti-Hermitian component could be removed, we can take another approach and subtract an approximation of eq 10 following the procedure by London^31,37,55^ which sets the coordinate **r** in the complex exponential be the midpoint between the two AOs
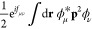
11which is both independent of the gauge-origin and Hermitian. This procedure leads to the Hamiltonian matrix elements
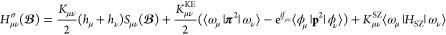
12where we have included some additional fitting parameters *K*_μν_^KE^ and *K*_μν_^SZ^ and  are the LAO overlap integrals. The spin-Zeeman interaction contributions can be evaluated as
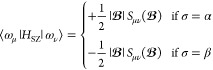
13for α and β spin electrons.

Considering these adaptions to extended Hückel theory, three modifications are identified which could be applied to any suitable zero-field semiempirical method.

- The basis set of AOs are multiplied by a field-dependent complex exponential to form a basis set of LAOs according to eq 8.
- Field-dependent kinetic energy correction terms are added to the effective one-electron Hamiltonian, as shown in eq 12.
- The spin-Zeeman interaction contributions are added to the effective one-electron Hamiltonian, as shown in eqs 12 and 13.

This approach ensures that the parent semiempirical approximation is recovered as . In addition, most existing terms in the semiempirical approach remain similar but are simply evaluated using LAOs in place of the original AOs. The additional contributions require only overlap and kinetic energy integrals over the LAOs to be evaluated, see, e.g., refs (15) and (16) for details of how to evaluate these contributions.

### 2.3 Density-Functional Tight-Binding Methods

In recent years, the GFN-xTB family of methods,^35,36,54^ developed for fast calculations of geometries, frequencies, and noncovalent interactions, has attracted significant attention owing to their broad but relatively simple parameterization for essentially the entire periodic table. The GFN-xTB methods are based on density-functional tight-binding approaches, but unlike earlier DFTB^46−49^ methods, the parameterization mostly avoids element pair-specific parameters. The GFN-xTB models are, therefore, excellent candidates to apply the modifications of Section 2.2.1 in practice, being relatively straightforward to implement and offering improved accuracy for a broad range of chemical applications.

Here, we focus on the widely available GFN1-xTB method,^35^ which we have implemented into our electronic structure program QUEST.^27^ Here, we use the same notation as given in ref (35) and refer the reader to this reference for further details. The GFN1-xTB energy is formed from a sum of the electronic (el), atom pairwise repulsion (rep), dispersion (disp), and halogen-bonding (XB) terms
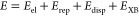
14We begin by making the assumption that, of these energy contributions, the electronic energy will undergo the most significant change when a magnetic field is applied and that all other terms can be approximated with their zero-field forms. By applying the modifications described in Section 2.2.1, we obtain the energy expression
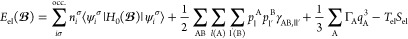
15Here, modifications to the first term of the expectation value of , the zero-order Hamiltonian, are made with the field-dependent contributions described below. The second and third terms are the self-consistent charge contributions. The second term depends on *p*_l_^A^, which are shell atomic charges, and γ_AB,ll′_, which describes the distance dependence of the Coulomb interaction according to the Mataga–Nishimoto–Ohno–Klopman^56−58^ formula. The summations are over atoms A and B and the atomic shells l(A), l′(B) on atoms A and B. In the third term, Γ_A_ is the charge derivative of the atomic Hubbard parameter and *q*_A_ is the Mulliken charge of atom A. The final term includes an electronic temperature, *T*_el_, times an electronic entropy, *S*_el_, and temperatures above 0K lead to fractional orbital occupations determined by Fermi smearing. See ref (35) for more details on each contribution.

Since the molecular orbitals, ψ_*i*_^σ^, are now formed from a linear combination of LAOs, the definition of the Mulliken and shell atomic charges employ the LAO overlap integrals. We, therefore, use a more general equation for the shell atomic charges for cases where the overlap and density matrices have nonvanishing imaginary components
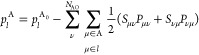
16where *S*_μν_ are the LAO overlap matrix elements and *P*_μν_ are the total density matrix elements.

The orbital coefficients are determined by solving the generalized eigenvalue problems
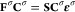
17where **F**^σ^ is the effective Kohn–Sham matrix. The elements of the Kohn–Sham matrix are given by the equation
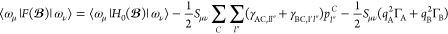
18where the zero-order Hamiltonian matrix elements are
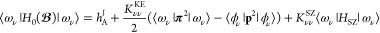
19for the diagonal terms and
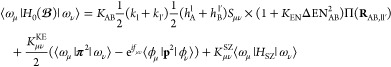
20for the off-diagonal terms where ΔEN_AB_ corresponds to the difference in electronegativities between atoms A and B and Π(**R**_AB,ll′_) is a distance and l-dependent function modulating this contribution—see ref (35) for further details.

### 2.4 Dual-Basis Approach

**Figure 1:**
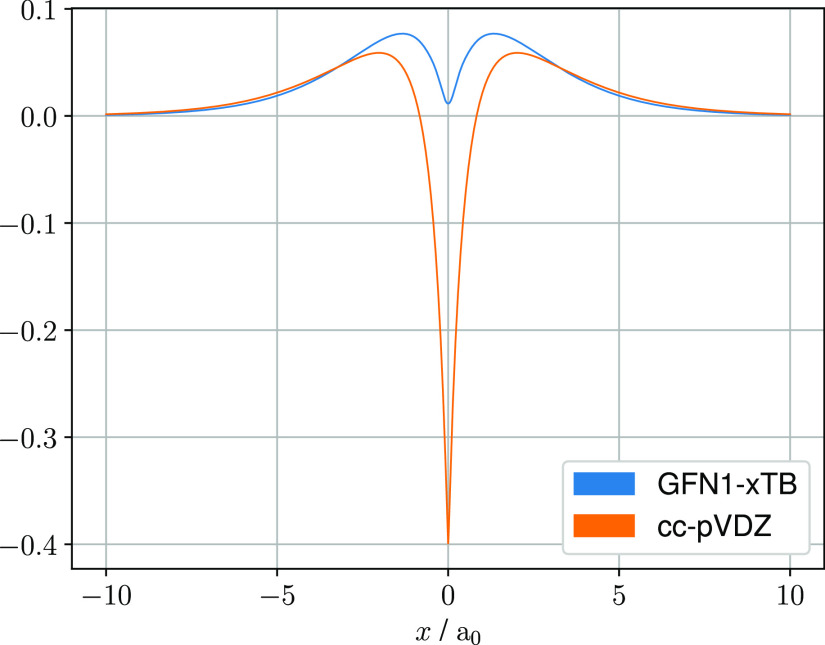
Values of the Li 2s basis function from the GFN1-xTB and cc-pVDZ basis sets plotted through the origin along the *x*-axis.

Considering the nature of the kinetic energy corrections introduced in eqs 12, 19, and 20, it is clear that the additional contributions due to the magnetic field corrections involve derivative operators. As a result, an adequate description of the nodal structure of the valence molecular orbitals is expected to have a significant bearing on the accuracy of the modified semiempirical approach as a function of the magnetic field strength. The specific choice of AOs may be regarded as a part of the parameterization of the semiempirical method. In the GFN1-xTB approach, a modest set of Slater-type AOs are selected, which are then approximated by Gaussian expansions with 4–6 primitives per AO. In the absence of a magnetic field, the energy expression depends only on overlap integrals involving the valence orbitals and so the basis functions used need not accurately describe the nodal structure of the AOs in the vicinity of each atom. This point is illustrated in Figure 1 where the lithium 2s basis function used in the GFN1-xTB parameterization is plotted and compared with a similar function from the larger cc-pVDZ basis set. It is clear that the GFN1-xTB basis function is accurate in the valence and outer valence regions but lacks the correct nodal structure.

To alleviate this issue, several avenues could be explored. The parameters *K*_μν_^KE^ could be used to improve the fit to calculated or experimental results. However, this may prove difficult since the underlying basis functions lack the desired physical structure. Alternatively, the entire basis set used in GFN1-xTB could be adjusted. However, since all other parameters in the approach are determined for a specific choice of basis set, this would require extensive efforts to reparameterize the method. Furthermore, the result would then be an approach that does not recover the established GFN1-xTB method in the absence of a magnetic field.

In the present work, we choose to explore a third alternative, namely, to (optionally) use a secondary basis set for the kinetic energy integrals so that
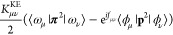
21becomes
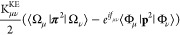
22where, in only the terms involving the **p**^2^ and **π**^2^ operators, we have replaced AOs and LAOs with a secondary set of basis functions (Φ_ν_ and Ω_ν_, respectively) that exhibit the correct nodal structure. As with the primary set of basis functions
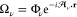
23so that the orbitals in zero and nonzero magnetic fields differ by a complex exponential. We will refer to the modified GFN1-xTB methods, which include an external magnetic field as the GFN1-xTB-M methods and their implementation with eqs 21 and 22 with the parameters set so that *K*_μν_^KE^ = *K*_μν_^SZ^ = 1 as the GFN1-xTB-M0 and GFN1-xTB-M1 methods, respectively.

In the present work, for the secondary set of LAOs in the kinetic energy integrals for the GFN1-xTB-M1 calculations, we used a subset of the basis functions from the cc-pVDZ basis set, except for the hydrogen atom which uses the original GFN1-xTB AOs. The basis functions from the cc-pVDZ basis set were chosen to have the correct number of nodes present for a given principal quantum number of that orbital; additionally, the signs of the contraction coefficients were changed to match with the phase of the GFN1-xTB basis functions; for details see the Supporting Information.

### 2.5 Translational Invariance and Geometrical Derivatives

Individual integrals over LAOs are not translationally invariant. In fact, upon translation of the basis function centers, the integrals change by a complex factor; for example, upon translation by **R**, the LAO overlap integrals change as
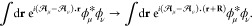
24

The role of the complex exponential in eq 11 is particularly important since it ensures that our energy expression remains translationally invariant since
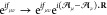
25which ensures, along with other terms, that the Hamiltonian and overlap integral matrix elements change by the same complex prefactor upon translation.

Key applications of semiempirical approaches are in structural optimization and exploration of potential energy surfaces. The low computational cost of such approaches means that they are often employed for preoptimization of structures prior to higher-level ab initio calculations and/or conformational searches and dynamics. Having established a translationally invariant and gauge-origin-independent expression for the energy in the presence of an external magnetic field, it is desirable to determine its analytic geometrical gradient to enable these types of studies. An advantage of the approach outlined in Section 2.2.1, which introduces minimal corrections to the parent semi-empirical method, is that similar, relatively simple, modifications are required to implement analytic geometrical gradients compared with the parent method. In particular, the main additional ingredients required are the LAO overlap and kinetic energy integral derivatives, which have been described and implemented in, e.g., ref (16). In the present work, we apply this implementation to construct the analytic derivatives of the GFN1-xTB-M approaches in the QUEST program.

## 3 Computational Methodology

GFN1-xTB-M calculations were carried out using QUEST^27^ with a default electronic temperature of 300 K using LAO integrals evaluated following the procedures outlined in ref (15). Molecular gradients were calculated analytically using LAO integral derivatives evaluated following the procedures in ref (16). GFN1-xTB-M magnetizabilities and NMR shielding constants were calculated using finite differences for the derivatives with respect to the magnetic field. In order to evaluate the performance of the GFN1-xTB-M approaches, comparisons were carried out with higher-level methods. For weak field properties, HF/STO-6G and HF/3-21G magnetizabilities and NMR shielding constants were calculated analytically using Dalton(59−62) and compared with previous benchmark coupled-cluster singles, doubles, and perturbative triples [CCSD(T)] data from the literature.^63,64^

For stronger fields, HF geometry optimizations and current density calculations were carried out using QUEST with the *u*-aug-cc-pCVTZ basis set^65−70^ (using the *u*-prefix to denote the uncontracted forms of the basis sets) for boron monohydride (BH), the *u*-aug-cc-pVDZ basis set^65−69^ for benzene, and the cc-pVDZ basis set^65−68,71−77^ for cyclooctatetraene (COT) and infinitene. Density functional theory (DFT) geometry optimizations of COT were also carried out using QUEST with the cTPSS exchange–correlation functional^9,78^ in the cc-pVDZ basis set. The resolution-of-the-identity (RI) approximation of the two-electron integrals and derivative integrals^18^ was used for all ab initio benzene, COT, and infinitene calculations; the auxiliary basis sets were generated with the AutoAux algorithm.^79^ Geometry optimizations were carried out using a quasi-Newton method with a damped BFGS updating procedure.

The GFN1-xTB-M1 calculations use a secondary basis set of LAOs which have the correct nodal structures (see Section 2.4) to evaluate the kinetic energy corrections. As the current density operator also contains derivatives, a secondary basis set is used to determine GFN1-xTB-M1 current densities which are consistent with the current densities that appear in the Biot–Savart law when deriving the equations for the NMR shielding constants from the GFN1-xTB-M1 energy expression.

## 4 Results and Discussion

Relative timings for the GFN1-xTB-M methods compared with those for the GFN1-xTB and HF approaches are presented in the Supporting Information for the COT molecule. In brief, increased computational costs are incurred in the GFN1-xTB-M methods due to the additional kinetic energy integrals evaluated and the complex arithmetic required. However, the computational costs of the GFN1-xTB-M0 and GFN1-xTB-M1 methods were found to be only around 2.8 times the cost of the GFN1-xTB method. In comparison, calculations on the same molecule at the HF/3-21G level take 3900 times longer. The GFN1-xTB-M approaches, therefore, maintain the 2–3 orders of magnitude speedup often associated with the GFN1-xTB method compared with Hartree–Fock and DFT approaches. The performance of the GFN1-xTB-M methods in the presence of a magnetic field is assessed, first for weak fields relevant to the evaluation of spectroscopic magnetic properties in Section 4.1 and then in much stronger fields in Section 4.2.

### 4.1 Weak Magnetic Fields

To test the GFN1-xTB-M methods in weak magnetic fields, we calculate isotropic magnetizabilities and the ^1^H, ^13^C, ^15^N, ^17^O, and ^19^F isotropic NMR shielding constants for a set of 28 molecules and compare against HF/STO-6G, HF/3-21G, and CCSD(T) results extrapolated to the basis set limit (obtained from refs (63) and (64)). The STO-6G and 3-21G basis sets were chosen as they have a similar number of valence basis functions to GFN1-xTB. All data points for the GFN1-xTB-M and HF calculations are available in the Supporting Information.

#### 4.1.1 Magnetizabilities

The magnetizability tensor, ξ_αβ_, is a second-order magnetic property which can be calculated from the derivative of the energy with respect to the external magnetic field^80^
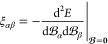
26where α and β denote Cartesian directions. Typically, the isotropic magnetizability is reported,  in SI units of 10^–30^ J T^–2^.

**Figure 2:**
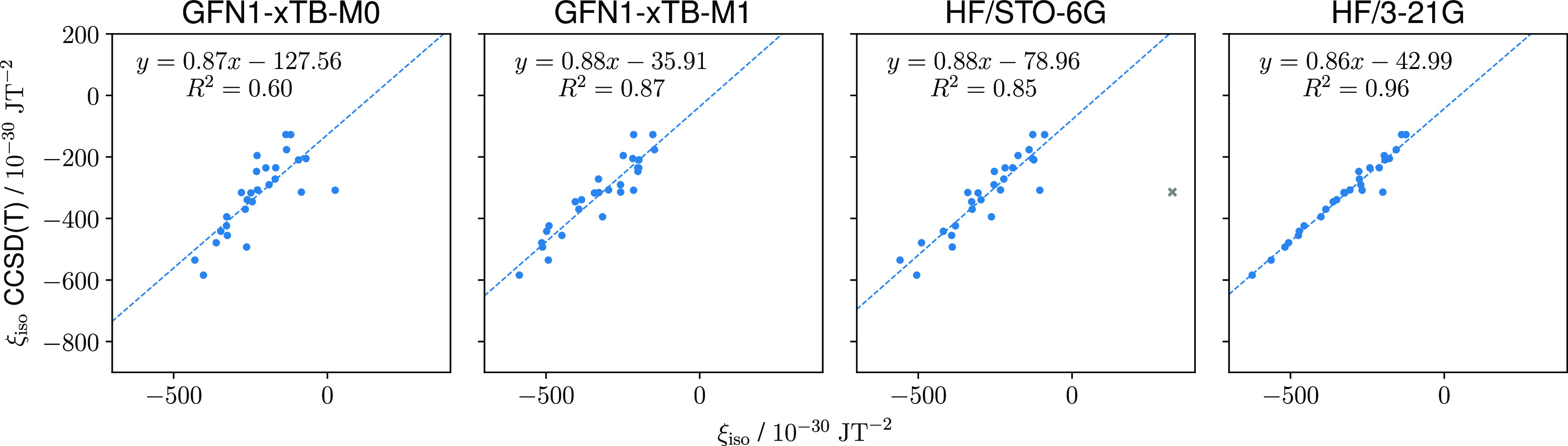
Correlation plots for GFN1-xTB-M0, GFN1-xTB-M1, HF/STO-6G, and HF/3-21G isotropic magnetizabilities with benchmark CCSD(T) data for all molecules in the test set except for O_3_. SO_2_ has been marked as a gray cross in the HF/STO-6G correlation plot and was not included in the linear regression analysis. CCSD(T) values were extrapolated to the basis set limit and were obtained from ref (63).

Since the GFN1-xTB approaches are valence-only models, it is not expected that absolute values of all properties should be quantitatively comparable with higher-level ab initio calculations or experimental measurements. However, if core-electron contributions to a given property are relatively system-independent, then one may expect chemical trends to be well reproduced. To assess this for the semiempirical calculation of isotropic magnetizabilities, we present correlation plots and linear regressions in Figure 2 to show the correlation between the GFN1-xTB-M results and benchmark CCSD(T) data; small-basis HF results are also included for comparison. As the HF magnetizability for O_3_ is particularly poor, owing to the multireference nature of this system, we have removed it from the correlation plots and linear regression analysis for all methods. Additionally for HF/STO-6G, SO_2_ magnetizabilities were also found to be particularly poor and so have been removed from the linear regression analysis for that method.

By examining the correlation plots in Figure 2, it is clear that the GFN1-xTB-M approaches offer a reasonable qualitative correlation with benchmark CCSD(T) calculations. From the *R*^2^ values of the linear regression analysis, the methods can be ranked in the order: GFN1-xTB-M0 < HF/STO-6G < GFN1-xTB-M1 < HF/3-21G. By comparing the first two panels of Figure 2, the benefit of the dual-basis GFN1-xTB-M1 approach is clear. GFN1-xTB-M0 yields magnetizabilities with large deviations from the CCSD(T) reference for several molecules, typically those containing heavier elements such as the H_2_S, HCP, OCS, PN, and SO_2_ molecules. This may be expected given the lack of nodal structure in the AOs (see Figure 1), and hence, for molecules with electrons occupying orbitals with higher principal quantum numbers, the errors are commensurately larger. By employing the GFN1-xTB-M1 method, the correct nodal structure of the orbitals is restored and the evaluation of the kinetic energy correction becomes more accurate, resulting in a substantial reduction in the errors for molecules with heavier atoms. Overall, the *R*^2^ value improves from 0.60 to 0.87 from GFN1-xTB-M0 to GFN1-xTB-M1 as a result.

To further assess the impact of the valence-only approximation in the GFN1-xTB-M approaches, it is interesting to compare with all-electron HF calculations in modest basis sets. Similar correlation plots are presented in Figure 2 for HF/STO-6G and HF/3-21G. Interestingly, HF/STO-6G performs worse than the GFN1-xTB-M1 method, which is unexpected given that HF is an all-electron ab initio method. However, closer inspection of the individual values (see the Supporting Information) suggests that this is likely due to the fact that the GFN1-xTB-M1 AOs include d-type functions for the third period p-block atoms, giving an increased flexibility that leads to improved results for the AlF, H_2_S, HCP, OCS, PN, and SO_2_ molecules over the HF/STO-6G results. As noted in ref (63), magnetizabilities are generally well described at the HF level and increasing the basis set flexibility slightly to the HF/3-21G level delivers a significantly improved correlation to the CCSD(T) results. Despite being formed from a smaller number of primitive Gaussian functions than STO-6G, the increased flexibility obtained by moving to a double-ζ basis set appears to lead to increased accuracy in the evaluation of the magnetizability. The 3-21G basis set still lacks the d-type functions for the third period p-block atoms which explains the poorer performance compared to GFN1-xTB-M1 for the sulfur-containing molecules H_2_S, OCS, and SO_2_.

Overall, the results for magnetizabilities are encouraging, particularly considering the minimal (physically motivated) implementation of the kinetic energy correction in the GFN1-xTB-M1 approach and that the underlying GFN1-xTB parameters had not been optimized for this property. The correlation plots show that the trends in the isotropic magnetizabilities can be reproduced reasonably accurately with this low-cost semiempirical approach.

#### 4.1.2 NMR Shielding Constants

Isotropic NMR shielding constants are another more challenging second-order magnetic property that can be calculated according to the derivative^85^
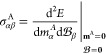
27where σ_αβ_^A^ is the NMR shielding tensor and *m*_α_^A^ is the magnetic dipole moment for nucleus A. The isotropic shielding is given by . The absolute values of isotropic NMR shielding constants have significant core contributions. However, these contributions are relatively system-independent for a given type of nucleus, see for example, the work by Gregor et al.,^81^ where the core contributions for C, Si, and P were estimated to be around 199, 832, and 902 ppm, respectively. As a consequence, it may be reasonable to expect valence-only semiempirical approximations to deliver results that correlate systematically with benchmark ab initio or experimental reference values. Indeed, previous attempts to apply semiempirical methods to the calculation of NMR properties^82−88^ suggest that qualitatively reasonable results can be obtained, particularly for ^1^H and ^13^C NMR. Furthermore, in practical applications, chemical shifts are typically calculated, relative to a given nucleus in a specific reference compound, leading to the cancellation of the core contributions.

**Figure 3:**
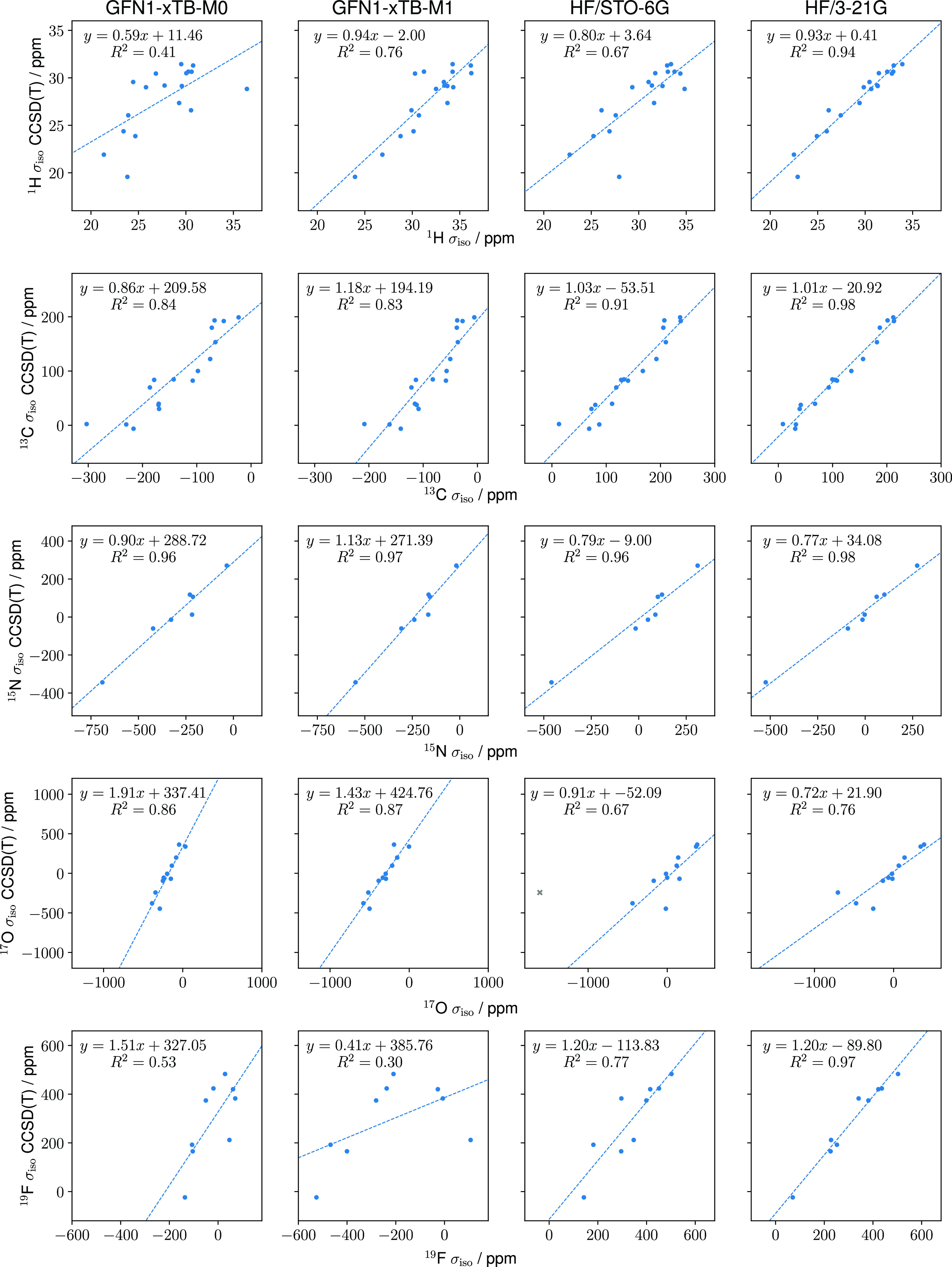
Correlation plots for GFN1-xTB-M0, GFN1-xTB-M1, HF/STO-6G, and HF/3-21G results with CCSD(T) benchmark data for ^1^H, ^13^C, ^15^N, ^17^O, and ^19^F isotropic NMR shielding constants for all molecules in the test set except for O_3_. SO_2_ has been marked as a gray cross in the HF/STO-6G correlation plot and was not included in the linear regression analysis. CCSD(T) values were extrapolated to the basis set limit and were obtained from ref (64).

To assess the ability of the GFN1-xTB-M methods to predict NMR shielding constants, a total of 18, 17, 7, 13, and 9 unique NMR shielding constants were calculated for the ^1^H, ^13^C, ^15^N, ^17^O, and ^19^F nuclei, respectively. Comparisons to benchmark CCSD(T) data from ref (64) and HF results in modest basis sets are shown in Figure 3. As we do not include the core contribution to the GFN1-xTB-M shielding constants, we only consider the correlation to CCSD(T) results for the set of NMR shielding constants with a given nucleus. Similar to the case for magnetizabilities, HF ^17^O NMR shielding constants for O_3_ are particularly poor and were removed from the analysis.

By examining Figure 3, it is clear that the correlation with benchmark CCSD(T) results is significantly better for ^1^H, ^13^C, and ^15^N NMR shielding constants than that for ^17^O and ^19^F. It is well known in the context of linear-response calculations at the DFT level that nuclei toward the top-right of the periodic table are more challenging for accurate NMR predictions. This is often traced to a stronger dependence on the paramagnetic component of the shielding constant, which depends sensitively on the occupied-virtual orbital energy differences.^66,89^ In this regard, it may be expected that ^15^N, ^17^O, and ^19^F NMR shielding predictions may be challenging for approaches using small basis sets, which limit the accuracy with which the virtual orbitals may be represented.

For ^1^H and ^13^C NMR shielding constants, similar observations may be compared to the behavior of the magnetizability calculations. Again, by comparing GFN1-xTB-M0 and GFN1-xTB-M1, we observe a systematic improvement for the latter, the dual-basis kinetic energy corrections, bringing the predictions more in-line with all-electron HF theory in modest basis sets. The intercepts from the linear regressions at the GFN1-xTB-M0 and GFN1-xTB-M1 levels quantify the systematic errors in the shielding constants. For the ^13^C NMR shielding constants, it is reassuring that the GFN1-xTB-M0 and GFN1-xTB-M1 intercepts of 209.6 and 194.2 ppm, respectively, bracket the core contribution of 199 ppm estimated by Gregor et al.^81^

For the ^15^N, ^17^O, and ^19^F NMR shielding predictions, the picture is more complex. The ^15^N predictions for GFN1-xTB-M0 and GFN1-xTB-M1 are surprisingly accurate, with *R*^2^ values close to HF calculations in STO-6G and 3-21G basis sets. Furthermore, the slope from the linear regression plots at the GFN1-xTB-M level is closer to the ideal value of 1 than that for the HF calculations. However, for the ^17^O and ^19^F nuclei, the predictions from both the GFN1-xTB-M and small basis HF calculations are relatively poor. For ^17^O, the GFN1-xTB-M *R*^2^ values are reasonable, but the slopes from the linear regressions are significantly too large. For the ^19^F NMR, the GFN1-xTB-M results have a poor correlation with CCSD(T) values, with significant scatter and low *R*^2^ values. For the ^15^N, ^17^O, and ^19^F nuclei, the correlation plots at the HF/STO-6G and HF/3-21G levels are also less impressive compared with those for ^1^H and ^13^C, consistent with their higher sensitivity to the description of the paramagnetic contributions and the poor representation of the virtual orbitals in modest basis sets.

Consistent with the observations for magnetizabilities, the choice of basis functions can also play a role in determining the quality of the results. For example, the ^17^O NMR shielding constant of SO_2_ is a significant outlier with errors of −1367.30 ppm in the STO-6G basis and −461.88 ppm in the 3-21G basis. The GFN1-xTB-M methods perform much better for this molecule, and this again is attributed to the inclusion of d-functions in the GFN1-xTB basis set parameterizations, which are absent in the STO-6G and 3-21G basis sets.

Overall, the performance in the NMR shielding constants is quite dependent on the nuclei considered. For ^1^H and ^13^C NMR, the correlation with CCSD(T) results for the GFN1-xTB-M1 method is good and the approach may provide a useful route to determine low-cost estimates of chemical shifts. Given that proton and carbon NMR are the most routinely used characterization techniques, the GFN1-xTB-M1 method is already a useful tool, particularly for larger systems where conventional ab initio calculations may be computationally demanding. However, for ^15^N, ^17^O, and ^19^F NMR, further investigations would be necessary to determine whether some reparameterization of the underlying GFN1-xTB parameters or the values of *K*_μν_^KE^ in the kinetic energy correction could overcome the intrinsic limitations of the modest basis sets employed, as has been attempted previously for older semiempirical methods.^82,83,87^

### 4.2 Strong Magnetic Fields

The modifications in the GFN1-xTB-M methods are designed to capture the necessary changes in the electronic Hamiltonian to describe systems under magnetic fields. Since these are incorporated in a nonperturbative manner, calculations can be performed at arbitrary field strengths. It is interesting therefore to consider to what extent these approaches may be applied in strong fields. The dominant corrections to the kinetic energy that modify the one-electron parts of the Hamiltonian are added explicitly and so should be well described. However, since we refrain from any reparameterization of the underlying GFN1-xTB model, one may expect to obtain (as the field strength increases) a poorer description of the effective electron–electron interactions. Since the GFN1-xTB-M1 method was shown to perform better for magnetizabilities and NMR shielding constants than its GFN1-xTB-M0 counterpart, we now test the range of applicability of this approach under strong magnetic fields. Field strengths up to 0.6*B*_0_ (*B*_0_ = ℏ e^–1^*a*_0_^–2^ = 2.3505 × 10^5^ T) are considered for the molecules BH, benzene, COT, and infinitene to test the extent to which GFN1-xTB-M1 can model exotic behaviors including para- to dia-magnetic transitions, changes in molecular structure and orientation, conformational preferences, and highly delocalized magnetically induced currents.

#### 4.2.1 Paramagnetic to Diamagnetic Transitions: BH

**Figure 4:**
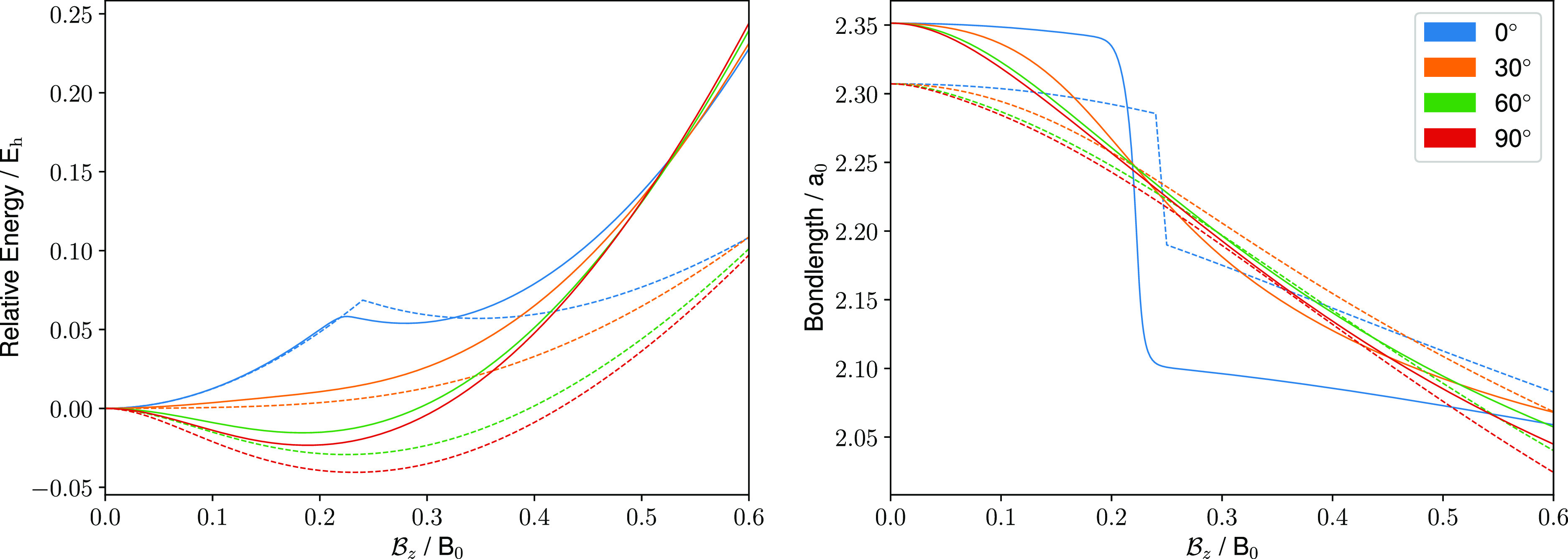
Relative energies and optimized bond lengths of BH for a range of magnetic field strengths with the magnetic field vector oriented along the *z*-axis and the BH bond-field angle constrained to values of 0, 30, 60, and 90°. Solid (dashed) lines show the relative energies and optimized bond lengths calculated with the GFN1-xTB-M1 (HF/*u*-aug-cc-pCVTZ) method.

The BH molecule is a prototypical system that has been extensively studied^1,2,90−93^ and exhibits both diamagnetism and (closed shell) paramagnetism depending on the orientation of the BH bond with respect to the magnetic field. It exhibits its most diamagnetic (paramagnetic) behavior with the bond aligned parallel (perpendicular) to the field. Furthermore, with orientations where the molecule is initially paramagnetic, a transition to diamagnetic behavior is observed with increased field strengths. This behavior is illustrated in the left-hand panel of Figure 4 where energies relative to the zero-field result are presented for a range of orientations at the GFN1-xTB-M1 (solid lines) and HF/*u*-aug-cc-pCVTZ (dashed lines) levels. For each field strength, the geometry of the molecule (with the orientation of the internuclear axis relative to the field constrained) has been optimized and the change in bond length as a function of the field for each orientation is shown in the right-hand panel.

The ability of GFN1-xTB-M1 to capture the qualitative behavior of the BH molecule as a function of the magnetic field strength is remarkable given the simplicity of the model. At the 0° orientation, the energy rises diamagnetically and a state crossing is observed just beyond , in close agreement with the much larger basis all-electron HF calculations. The relative energies also capture the preference for the 90° perpendicular orientation, along with the transition from paramagnetic to diamagnetic behavior at this orientation with increasing field strength. Overall, the GFN1-xTB-M1 model predicts that BH will exhibit diamagnetism for bond-field angles of 0 and 30° and paramagnetism for 60 and 90° at zero-field, in line with much more expensive ab initio calculations.

This qualitatively correct behavior suggests that to a large extent, the energetics are determined by the adequate treatment of the one-electron corrections in the presence of the magnetic field. This is further confirmed by the consideration of how the BH bond length changes as a function of the magnetic field in each orientation. Again, GFN1-xTB-M1 correctly captures the qualitative behavior, showing a bond length contraction with increasing field strengths in all orientations. In addition, the rate of decrease in the bond length is also qualitatively captured, with the initial slope in the right-hand panel of Figure 4 becoming more negative with an increasing angle between the internuclear axis and the magnetic field vector.

As expected, quantitative agreement between the GFN1-xTB-M1 and HF/*u*-aug-cc-pCVTZ results deteriorates for higher field strengths. For field strengths in the range , the agreement is remarkable, particularly given the significant approximations in the GFN1-xTB-M1 approach. Generally, the paramagnetic response to the field is underestimated, while the diamagnetic response is overestimated in the relative energies. This is particularly clear for , where the diamagnetic rise of the GFN1-xTB-M1 energies are much too steep relative to the HF results. Overall, the ability of GFN1-xTB-M1 to describe the strong field response is encouraging—predicting the paramagnetic to diamagnetic transition with increasing field strength and the preferred perpendicular orientation correctly. Since BH exhibits closed-shell paramagnetism, this indicates that the balance between the orbital-Zeeman and diamagnetic contributions is well described in the GFN1-xTB-M1 model.

#### 4.2.2 Molecular Structure and Orientation: Benzene

**Figure 5:**
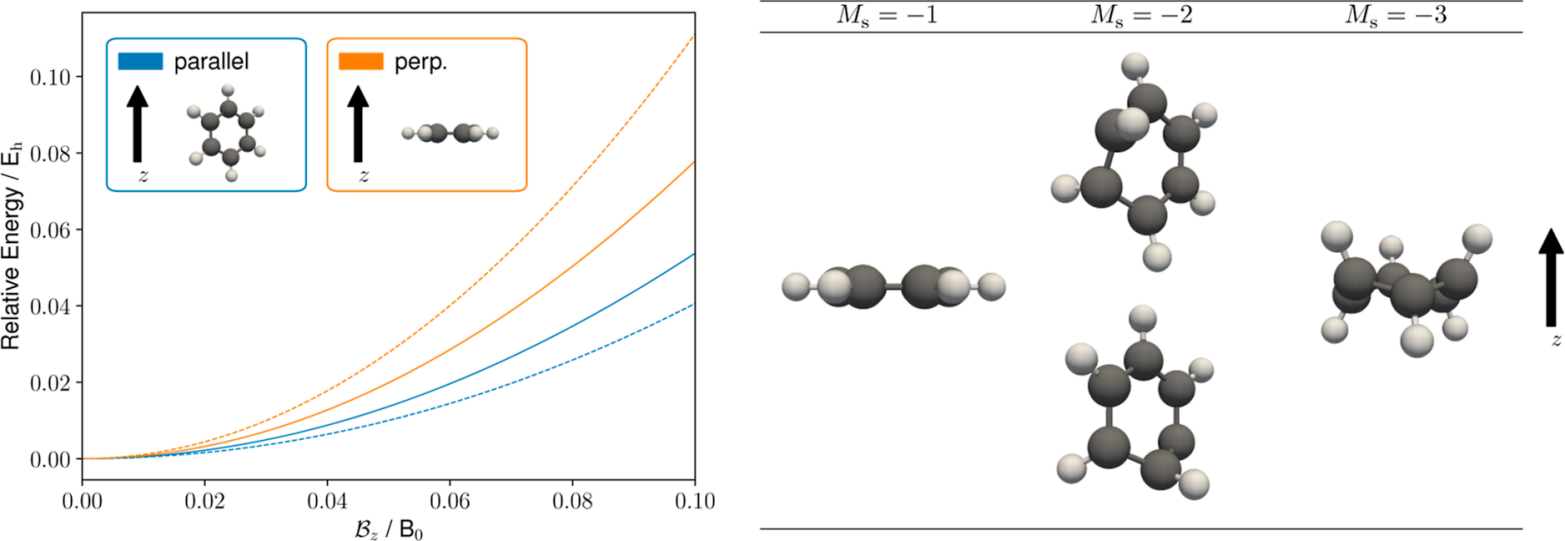
Left: relative energies of benzene (*M*_s_ = 0) for a range of magnetic fields with the magnetic field vector oriented along the *z*-axis with the plane of the benzene ring constrained to be parallel or perpendicular to the field vector. Solid (dashed) lines show the relative energies calculated with the GFN1-xTB-M1 (HF/*u*-aug-cc-pVDZ) method. Right: GFN1-xTB-M1 optimized molecular structures of benzene (*M*_s_ = −1, −2, and −3) under a magnetic field oriented along the *z*-axis with .

Recently, analytic gradients were implemented at the HF and CDFT levels for LAO basis sets in ref (16) and the benzene molecule was examined as a prototypical system to explore the structure of conjugated aromatic molecules in the presence of an external magnetic field. In Figure 5, the relative energy for the closed-shell *M*_s_ = 0 electronic configuration of benzene is presented at the equilibrium geometries for a range of magnetic fields with the plane of the ring constrained to be parallel or perpendicular to the field. As with the BH molecule, there is a good qualitative agreement between GFN1-xTB-M1 and HF. For both methods, there is an increase in energy with increasing field strength, as expected for this state. Additionally, in both cases, the lowest energy is obtained when the plane of the benzene ring is parallel to the magnetic field, again confirming that the GFN1-xTB-M1 method can capture the correct orientation with respect to the applied field. Here, we also note that the perpendicular orientation was considered for the *M*_s_ = 0 electronic configuration in ref (16), rather than the lower-energy parallel orientation.

The implementation of the GFN1-xTB-M1 method also allows for open-shell calculations, in which different *M*_s_ values may be selected and geometry optimizations can be carried out for each configuration. The GFN1-xTB-M1 approach produces a qualitatively similar energy profile to Figure 5 from ref (16) for the *M*_s_ = 0, −1, −2, and −3 configurations (0, 2, 4, and 6 unpaired β-spin electrons, respectively). As electrons are progressively unpaired with increasing *M*_s_, the spin-Zeeman interactions become more significant; as a result, each configuration with more unpaired β-spin electrons is driven down in energy, each becoming the ground state at specific field strengths before rising diamagnetically. In addition, each configuration has a qualitatively different molecular structure, varying from a planar hexagon (*M*_s_ = 0), to a distorted but planar hexagon (*M*_s_ = −1), to a half-chair-like conformation (*M*_s_ = −2), and to a chair-like conformation (*M*_s_ = −3). Furthermore, each configuration has a different preferred orientation with respect to an applied field. In the right-hand panel of Figure 5, we present the corresponding optimized GFN1-xTB-M1 structures with . Remarkably, not only are the alignments of each structure consistent with those presented in ref (16) at the HF and cTPSS levels, but similar conformations are also obtained. For the *M*_s_ = −1 configuration, a similarly distorted hexagonal structure is obtained. For *M*_s_ = −2, the GFN1-xTB-M1 method predicts two low-lying conformers, a half-chair (lower) and a more distorted structure (upper) which begins to resemble a more chair-like conformation. The GFN1-xTB-M1 method predicts that this more distorted structure is lower in energy by 2.4 × 10^–4^ E_h_, while the cTPSS calculations favor the half-chair conformation. Performing a cTPSS optimization in the same basis set used in ref (16) from the more distorted structure yields a similarly distorted conformation, 1.6 × 10^–4^ E_h_ above the half-chair conformation. This indicates that care should be taken when searching for equilibrium structures in the presence of an external field and that GFN1-xTB-M1 may be a useful tool for identifying different candidates for the lowest-energy conformers. For the *M*_s_ = −3 configuration, the GFN1-xTB-M1 method predicts a chair conformation as the lowest energy, in line with results at the HF and cTPSS levels.

The results for benzene suggest that the GFN1-xTB-M1 method may be a valuable tool for preoptimizations of molecular structures in the presence of a strong magnetic field. Indeed, when performing geometry optimizations in a field, a substantial number of optimization cycles are related to the molecule aligning to a preferred orientation—it is therefore useful to have low-cost approaches capable of finding these orientations relatively quickly.

#### 4.2.3 Conformational Preference in a Magnetic Field: COT

Molecules under a magnetic field can favor specific orientations, as seen for the BH and benzene calculations. Conformer searches, therefore, are more challenging due to the additional degrees of freedom necessary to describe the molecule’s orientation relative to the magnetic field. A magnetic field could also stabilize conformers not observed at zero-field. The conformational preferences of COT, which in its planar conformation is often considered as an archetypal antiaromatic system,^94^ are examined using GFN1-xTB-M1 and the results are compared with HF and cTPSS calculations in the cc-pVDZ basis.

**Figure 6:**
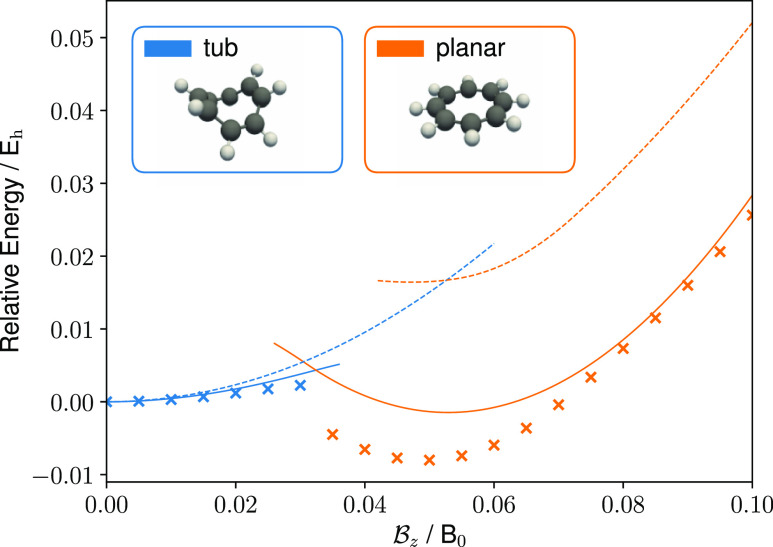
Relative energies of COT for the tub and planar conformers for a range of magnetic field strengths with the magnetic field vector oriented along the *z*-axis. Solid (dashed) lines show the relative energies calculated with the GFN1-xTB-M1 (HF/cc-pVDZ) method, and crosses show the relative energies calculated using the DFT with the cTPSS/cc-pVDZ exchange–correlation functional and basis set.

Energy minimizations were carried out for COT using initial structures in the planar and tub conformations for a range of magnetic field strengths to determine the energetically preferred conformations. The results are presented in Figure 6, where energies are shown relative to the optimized tub conformer in the absence of a field. For both GFN1-xTB-M1 and HF, we see the stabilization (destabilization) of the planar (tub) conformer at higher field strengths, with the planar conformer eventually becoming the lower energy conformer at a field strength of  for GFN1-xTB-M1 and  for HF. Although GFN1-xTB-M1 correctly predicts the stabilization of the planar conformer with increasing field strength, the relative energies of this conformer at higher fields appear to be significantly lower than those for HF.

To investigate the effect of the electron correlation on the relative energies of the optimized geometries, the tub and planar conformers from the HF calculations were reoptimized at the cTPSS/cc-pVDZ level with the RI approximation; the resulting relative energies are shown as crosses in Figure 6. Interestingly, the GFN1-xTB-M1 results are closer to the cTPSS values, with both approaches predicting an earlier stabilization of the planar conformer compared to HF calculations.

The preferred conformations of the COT molecule as a function of the field in the closed-shell *M*_s_ = 0 configuration considered in Figure 6 can be understood in terms of the contributions to the electronic Hamiltonian arising from the magnetic field. Initially, the molecule adopts a tub conformation; as the field strength increases, the tub aligns predominantly perpendicular to the field and then begins to flatten. This is consistent with the orbital-Zeeman contribution becoming more significant. Beyond a critical field strength, the minimum corresponding to the planar conformation is more stable than the tub conformation. Initially, the planar conformation aligns perpendicular to the field, maximizing the stabilization due to the orbital-Zeeman contribution (which is linear in the field strength). However, with stronger field strengths, the diamagnetic contribution (which is quadratic in the field strength) becomes more significant and a planar conformation that is tilted relative to the field is adopted to balance the stabilization effects from the orbital angular momenta while reducing the molecular volume perpendicular to the magnetic field to offset the diamagnetic contribution. Eventually, with even stronger field strengths, the diamagnetic terms dominate and a more parallel alignment with the field vector is adopted. The GFN1-xTB-M1 method correctly captures this behavior in good qualitative agreement with the much more computationally demanding HF and cTPSS calculations.

#### 4.2.4 Magnetically Induced Currents: Benzene and Infinitene

Magnetically induced currents are often calculated to aid in the interpretation of NMR chemical shifts and as a tool to probe electron delocalization and hydrogen bonding.^95^ Often, response currents are calculated using quantities available from codes that perform ab initio NMR calculations.^96^ However, they may also be calculated directly in nonperturbative calculations as a function of the applied magnetic field; see for example ref (30).

**Figure 7:**
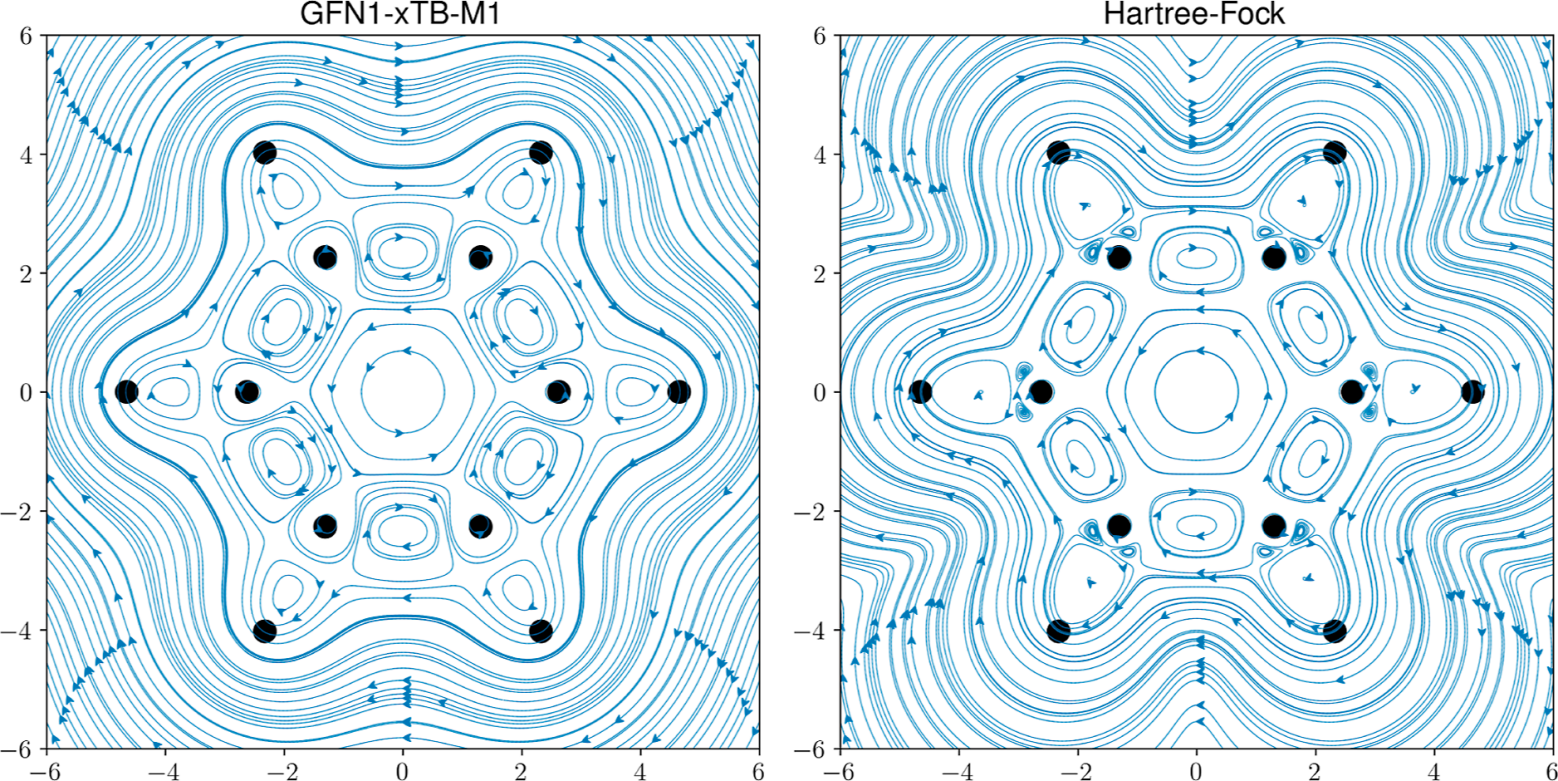
Streamline plots of the GFN1-xTB-M1 and HF/*u*-aug-cc-pVDZ current densities of benzene with the closed-shell *M*_s_ = 0 electronic configuration under a magnetic field oriented along the *z*-axis with . The benzene molecule lies in the *xy*-plane, and streamlines are plotted across the molecular plane.

**Figure 8:**
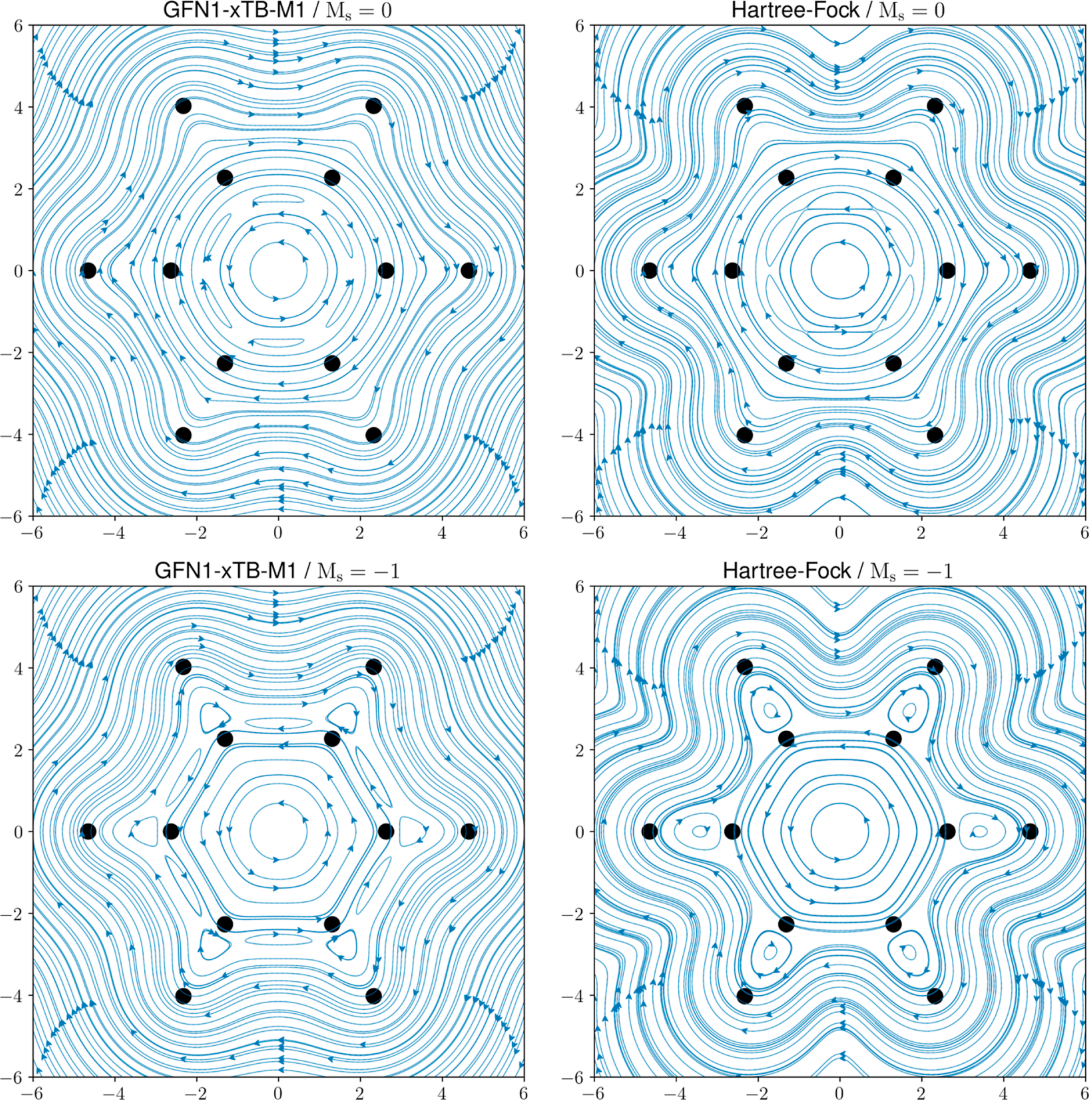
Streamline plots of the GFN1-xTB-M1 and HF/*u*-aug-cc-pVDZ current densities of benzene with the closed-shell *M*_s_ = 0 and open-shell *M*_s_ = −1 electronic configurations under a magnetic field oriented along the *z*-axis with . The benzene molecule lies in the *xy*-plane, and streamlines were generated at a height of 1.0a_0_ above the molecular plane.

To assess the quality of magnetically induced currents from the GFN1-xTB-M1 method, we begin by considering the archetypal benzene molecule ring current in the *M*_s_ = 0 electronic configuration under a magnetic field of 0.1*B*_0_ perpendicular to the molecular plane. Since GFN1-xTB-M1 is a valence-only model, some differences in the current densities are expected due to the omitted carbon 1s electrons. In Figure 7, streamlines are plotted across the molecular plane for the current densities for the closed-shell *M*_s_ = 0 electronic configuration; the open-shell *M*_s_ = −1 current densities are very similar; see the Supporting Information. In Figure 8, the current densities are also shown at 1.0 a_0_ above the molecular plane, highlighting the π-delocalization.

From Figure 7, it is clear that the HF current densities exhibit a larger number of features than those of GFN1-xTB-M1. For example, the small vortices near the carbon atoms present in the HF current densities are absent in the GFN1-xTB-M1 current densities. However, more important features representing valence electron delocalization are reproduced well by GFN1-xTB-M1, such as the diatropic (paratropic) ring currents outside (inside) of the carbon ring and the vortices between the carbon and hydrogen atoms. Furthermore, away from the molecular plane, in Figure 8, even greater similarities between the GFN1-xTB-M1 and HF methods are apparent for both the *M*_s_ = 0 and *M*_s_ = −1 electronic configurations since the current density features close to the carbon nuclei are not visible. As we move from a height of 0.0 to 1.0a_0_, we also see that the GFN1-xTB-M1 method correctly reproduces the contraction (expansion) in the paratropic current for the *M*_s_ = 0 (*M*_s_ = −1) electronic configurations.

Infinitene was first synthesized by Krzeszewki et al. and consists of 12 benzene rings fused in a loop resembling an infinity symbol,^97^ which has the structure of a topological cylinder with one full twist, a half twist more than a Möbius strip. Unlike a Möbius strip, which has one edge and one side, infinitene has two edges and two sides but, like the Möbius strip, will have a chirality depending on the direction of the twist in its structure, leading to the (P,P)-infinitene and (M,M)-infinitene enantiomers. From the streamline plots of the current density of benzene, it was observed that there were currents flowing around the edges of the benzene ring; one might therefore expect currents to form in infinitene which flow along its two edges.

Using CAM-B3LYP/def2-SVP with the gauge-including magnetically induced current method,^95,96,98^ Orozco-Ic et al. showed computationally that, under a magnetic field, there are two sets of nonintersecting currents in infinitene that flow along its edges.^99^ As GFN1-xTB-M1 current densities exhibited good qualitative agreement with ab initio calculations for benzene, infinitene is used to further test GFN1-xTB-M1 with larger nonplanar molecules.

**Figure 9:**
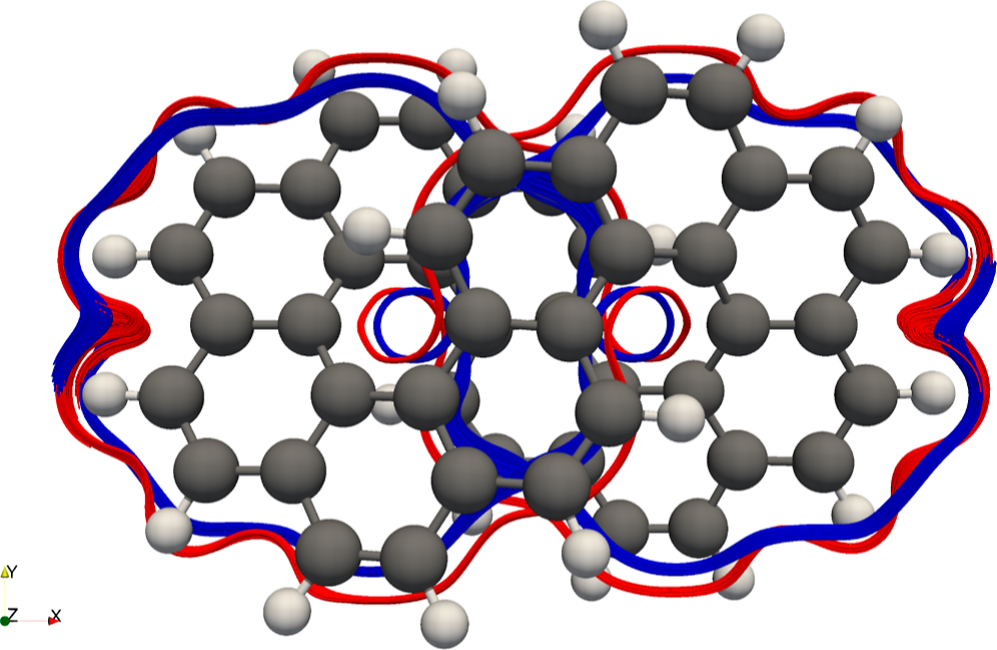
Streamlines generated for the GFN1-xTB-M1 and HF/cc-pVDZ current densities of (P,P)-infinitene under a magnetic field oriented along the *z*-axis with  using ParaView.^101^ GFN1-xTB-M1 streamlines (blue) were generated using a point cloud seed type with a radius of 0.1 centered at (±4.5, 0.0, 0.0), and HF streamlines (red) were generated using a point cloud seed type with a radius of 0.1 centered at (±5.0, 0.0, 0.0).

In Figure 9, we show the magnetically-induced current density streamlines visualized using ParaView^100,101^ from GFN1-xTB-M1 and HF current densities of (P,P)-infinitene in the presence of a magnetic field with . (P,P)-infinitene geometries were taken from ref (99), and streamlines were seeded from (±4.5, 0.0, 0.0) for GFN1-xTB-M1 and (±5.0, 0.0, 0.0) for HF. The GFN1-xTB-M1 method reproduces the nonintersecting currents remarkably well, going around or above the edges of the infinitene molecule in agreement with HF results here and the response currents in ref (99).

## 5 Conclusions

A general approach to modify semiempirical methods so that the effects of a strong magnetic field can be included has been presented. The approach was applied to the density-functional tight-binding method GFN1-xTB, leading to the GFN1-xTB-M methods which include field-dependent kinetic energy corrections and spin-Zeeman interaction terms. To improve the description of the kinetic energy corrections, a dual-basis approach, GFN1-xTB-M1, was introduced, in which all terms evaluated over derivative operators employed a secondary LAO basis set. This secondary set was constructed to capture the correct nodal structure of the AOs, missing in the original GFN1-xTB basis.

The performance of the GFN1-xTB-M1 method was benchmarked in weak magnetic fields for the calculation of magnetic properties, comparing with a single basis approach GFN1-xTB-M0 as well as HF calculations in modest basis sets and accurate large basis CCSD(T) benchmark data. The dual-basis GFN1-xTB-M1 method was consistently found to significantly outperform the single-basis GFN1-xTB-M0 method, often being competitive with all-electron HF calculations in modest basis sets and correlating well with large basis set CCSD(T) benchmark data for magnetizabilities. Similar observations were made for ^1^H and ^13^C NMR shielding calculations, where GFN1-xTB-M1 offers good performance. However, for ^15^N, ^17^O, and ^19^F NMR, the small basis of LAOs used in the GFN1-xTB-M approaches may limit the accuracy with which the paramagnetic component of the shielding constants may be described. Small basis HF calculations also suffer from this deficiency and also exhibit poor accuracy compared with large basis set CCSD(T) benchmark data.

The range of applicability of the GFN1-xTB-M1 method in strong magnetic fields was explored. Remarkably good performance was observed in comparison with ab initio calculations, with GFN1-xTB-M1 qualitatively reproducing a wide range of exotic phenomena including para- to dia-magnetic transitions with BH, preferential orientation of the molecular frame with respect to the magnetic field with BH, benzene and COT, the preferred molecular structures of different electronic configurations of benzene in a strong field, the transition from tub to planar conformation of COT with increasing field, and the structure of the magnetically induced currents in benzene and infinitene. Taken together, these observations suggest that GFN1-xTB-M1 may be a useful tool in its own right for studies of large systems in the presence of strong magnetic fields and the rapid exploration of the more complex potential energy surfaces encountered under these conditions. Furthermore, the ability of the approach to determine reasonable structures for energetically low-lying conformers suggests that it may be a useful preoptimization/prescreening tool for subsequent, more expensive, ab initio calculations.

Throughout the present study, no reparameterizations of the quantities defining the GFN1-xTB-M model in the absence of a field were carried out, ensuring that the GFN1-xTB-M methods reduce to the parent GFN1-xTB method. Interestingly, the observation that the correct qualitative behavior is obtained for a wide range of exotic properties for  studied in the present work suggests that the one-electron kinetic energy corrections and spin-Zeeman contributions, which are explicitly treated in the models presented, are the dominant contributions that govern the behavior. However, it is also clear that quantitative agreement with ab initio data deteriorates for , suggesting that for larger fields, reparameterization may be beneficial.

The GFN1-xTB-M methods provide a useful tool for the study of magnetic properties and the exploration of potential energy surfaces and reactivity in strong magnetic fields, with applications similar to the parent GFN1-xTB method. Work is ongoing to explore how the computational efficiency of these methods can be used to study electronic^13^ and nuclear^102−106^ dynamics in the presence of magnetic fields, opening the way to calculate a wider range of combined electronic, vibrational, and magnetic spectroscopic properties directly at the semiempirical level. Future work will consider how the accuracy of these approaches may be further refined. Several directions could be pursued in this regard. New parameters could be determined to redefine the underlying models—many of which are fitted to calculated ab initio data and field-dependent quantities for reparameterization could be readily calculated; see, e.g., ref (20). More sophisticated semiempirical methods could be adapted in the same manner as described in the present work, such as the GFN2-xTB method.^36,54^ In addition, the underlying basis sets employed in the semiempirical approach could be reoptimized, ensuring correct nodal structure in the underlying AOs. The secondary basis sets used in this work represent only a first step in this direction. Finally, the extended approaches presented here could form a basis for enhanced machine-learning models, such as those presented in refs (107) and (108).

## Supporting Information Available

Full results of the GFN1-xTB-M and HF magnetic property calculations (PDF)
Basis set file of the secondary set of basis functions used by the GFN1-xTB-M1 method to evaluate the kinetic energy integrals (TXT)

## References

[ref1] Tellgren E. I.; Soncini A.; Helgaker T. Nonperturbative *ab initio* calculations in strong magnetic fields using London orbitals. J. Chem. Phys. 2008, 129, 154114 10.1063/1.2996525. 19045183

[ref2] Tellgren E. I.; Helgaker T.; Soncini A. Non-perturbative magnetic phenomena in closed-shell paramagnetic molecules. Phys. Chem. Chem. Phys. 2009, 11, 5489 10.1039/b822262b. 19551219

[ref3] Lange K. K.; Tellgren E. I.; Hoffmann M. R.; Helgaker T. A Paramagnetic Bonding Mechanism for Diatomics in Strong Magnetic Fields. Science 2012, 337, 327–331. 10.1126/science.1219703. 22822146

[ref4] Tellgren E. I.; Kvaal S.; Sagvolden E.; Ekström U.; Teale A. M.; Helgaker T. Choice of basic variables in current-density-functional theory. Phys. Rev. A: At., Mol., Opt. Phys. 2012, 86, 062506 10.1103/physreva.86.062506.

[ref5] Tellgren E. I.; Teale A. M.; Furness J. W.; Lange K. K.; Ekström U.; Helgaker T. Non-perturbative calculation of molecular magnetic properties within current-density functional theory. J. Chem. Phys. 2014, 140, 034101 10.1063/1.4861427. 25669357

[ref6] Stopkowicz S.; Gauss J.; Lange K. K.; Tellgren E. I.; Helgaker T. Coupled-cluster theory for atoms and molecules in strong magnetic fields. J. Chem. Phys. 2015, 143, 074110 10.1063/1.4928056. 26298118

[ref7] Sun S.; Williams-Young D. B.; Stetina T. F.; Li X. Generalized Hartree–Fock with Nonperturbative Treatment of Strong Magnetic Fields: Application to Molecular Spin Phase Transitions. J. Chem. Theory Comput. 2018, 15, 348–356. 10.1021/acs.jctc.8b01140. 30485745

[ref8] Sun S.; Williams-Young D.; Li X. An ab Initio Linear Response Method for Computing Magnetic Circular Dichroism Spectra with Nonperturbative Treatment of Magnetic Field. J. Chem. Theory Comput. 2019, 15, 3162–3169. 10.1021/acs.jctc.9b00095. 30933558

[ref9] Furness J. W.; Verbeke J.; Tellgren E. I.; Stopkowicz S.; Ekström U.; Helgaker T.; Teale A. M. Current Density Functional Theory Using Meta-Generalized Gradient Exchange-Correlation Functionals. J. Chem. Theory Comput. 2015, 11, 4169–4181. 10.1021/acs.jctc.5b00535. 26575912

[ref10] Hampe F.; Stopkowicz S. Equation-of-motion coupled-cluster methods for atoms and molecules in strong magnetic fields. J. Chem. Phys. 2017, 146, 154105 10.1063/1.4979624. 28433009

[ref11] Hampe F.; Gross N.; Stopkowicz S. Full triples contribution in coupled-cluster and equation-of-motion coupled-cluster methods for atoms and molecules in strong magnetic fields. Phys. Chem. Chem. Phys. 2020, 22, 23522–23529. 10.1039/d0cp04169f. 33078770

[ref12] Reimann S.; Borgoo A.; Austad J.; Tellgren E. I.; Teale A. M.; Helgaker T.; Stopkowicz S. Kohn–Sham energy decomposition for molecules in a magnetic field. Mol. Phys. 2018, 117, 97–109. 10.1080/00268976.2018.1495849.

[ref13] Wibowo M.; Irons T. J. P.; Teale A. M. Modeling Ultrafast Electron Dynamics in Strong Magnetic Fields Using Real-Time Time-Dependent Electronic Structure Methods. J. Chem. Theory Comput. 2021, 17, 2137–2165. 10.1021/acs.jctc.0c01269. 33724806 PMC8047917

[ref14] David G.; Irons T. J. P.; Fouda A. E. A.; Furness J. W.; Teale A. M. Self-Consistent Field Methods for Excited States in Strong Magnetic Fields: a Comparison between Energy- and Variance-Based Approaches. J. Chem. Theory Comput. 2021, 17, 5492–5508. 10.1021/acs.jctc.1c00236. 34517708

[ref15] Irons T. J. P.; Zemen J.; Teale A. M. Efficient Calculation of Molecular Integrals over London Atomic Orbitals. J. Chem. Theory Comput. 2017, 13, 3636–3649. 10.1021/acs.jctc.7b00540. 28692291

[ref16] Irons T. J. P.; David G.; Teale A. M. Optimizing Molecular Geometries in Strong Magnetic Fields. J. Chem. Theory Comput. 2021, 17, 2166–2185. 10.1021/acs.jctc.0c01297. 33724812 PMC8047810

[ref17] Pemberton M. J.; Irons T. J. P.; Helgaker T.; Teale A. M. Revealing the exotic structure of molecules in strong magnetic fields. J. Chem. Phys. 2022, 156, 204113 10.1063/5.0092520. 35649858

[ref18] Pausch A.; Klopper W. Efficient evaluation of three-centre two-electron integrals over London orbitals. Mol. Phys. 2020, 118, e1736675 10.1080/00268976.2020.1736675.

[ref19] Wibowo M.; Huynh B. C.; Cheng C. Y.; Irons T. J. P.; Teale A. M. Understanding ground and excited-state molecular structure in stong magnetic fields using the maximum overlap method. Mol. Phys. 2022, 121, e2152748 10.1080/00268976.2022.2152748.

[ref20] Francotte R.; Irons T. J. P.; Teale A. M.; de Proft F.; Geerlings P. Extending conceptual DFT to include external variables: the influence of magnetic fields. Chem. Sci. 2022, 13, 5311–5324. 10.1039/d1sc07263c. 35655570 PMC9093152

[ref21] Irons T. J. P.; Huynh B. C.; Teale A. M.; De Proft F.; Geerlings P. Molecular charge distributions in strong magnetic fields: a conceptual and current DFT study. Mol. Phys. 2022, e2145245 10.1080/00268976.2022.2145245.

[ref22] Holzer C.; Teale A. M.; Hampe F.; Stopkowicz S.; Helgaker T.; Klopper W. GW quasiparticle energies of atoms in strong magnetic fields. J. Chem. Phys. 2019, 150, 214112 10.1063/1.5093396. 31176321

[ref23] Pausch A.; Gebele M.; Klopper W. Molecular point groups and symmetry in external magnetic fields. J. Chem. Phys. 2021, 155, 201101 10.1063/5.0069859. 34852467

[ref24] LONDON, A quantum chemistry program for plane–wave/GTO hybrid basis sets and finite magnetic field calculations. http://londonprogram.org.

[ref25] BAGEL, Brilliantly Advanced General Electronic-Structure Library. http://nubakery.org; Published under the GNU General Public License.

[ref26] TURBOMOLE V6.2; A development of University of Karlsruhe and Forschungszentrum Karlsruhe GmbH, 1989–2007, TURBOMOLE GmbH, 2010. http://turbomole.com.

[ref27] QUEST, A Rapid Development Platform for QUantum Electronic Structure Techniques. https://quest.codes/.

[ref28] Speake B. T.; Irons T. J. P.; Wibowo M.; Johnson A. G.; David G.; Teale A. M. An Embedded Fragment Method for Molecules in Strong Magnetic Fields. J. Chem. Theory Comput. 2022, 18, 7412–7427. 10.1021/acs.jctc.2c00865. 36414537 PMC9753591

[ref29] Holzer C.; Pausch A.; Klopper W. The GW/BSE Method in Magnetic Fields. Front. Chem. 2021, 9, 746162 10.3389/fchem.2021.746162. 34900932 PMC8655096

[ref30] Irons T. J. P.; Spence L.; David G.; Speake B. T.; Helgaker T.; Teale A. M. Analyzing Magnetically Induced Currents in Molecular Systems Using Current-Density-Functional Theory. J. Phys. Chem. A 2020, 124, 1321–1333. 10.1021/acs.jpca.9b10833. 31986039

[ref31] London F. Théorie quantique des courants interatomiques dans les combinaisons aromatiques. J. Phys. Radium 1937, 8, 397–409. 10.1051/jphysrad:01937008010039700.

[ref32] Ceulemans A. J. Group Theory Applied to Chemistry; Springer Netherlands, 2013.

[ref33] Blaschke S.; Stopkowicz S. Cholesky decomposition of complex two-electron integrals over GIAOs: Efficient MP2 computations for large molecules in strong magnetic fields. J. Chem. Phys. 2022, 156, 044115 10.1063/5.0076588. 35105060

[ref34] Gauss J.; Blaschke S.; Burger S.; Nottoli T.; Lipparini F.; Stopkowicz S. Cholesky decomposition of two-electron integrals in quantum-chemical calculations with perturbative or finite magnetic fields using gauge-including atomic orbitals. Mol. Phys. 2022, 121, e2101562 10.1080/00268976.2022.2101562.

[ref35] Grimme S.; Bannwarth C.; Shushkov P. A Robust and Accurate Tight-Binding Quantum Chemical Method for Structures, Vibrational Frequencies, and Noncovalent Interactions of Large Molecular Systems Parametrized for All spd-Block Elements (Z = 1–86). J. Chem. Theory Comput. 2017, 13, 1989–2009. 10.1021/acs.jctc.7b00118. 28418654

[ref36] Bannwarth C.; Caldeweyher E.; Ehlert S.; Hansen A.; Pracht P.; Seibert J.; Spicher S.; Grimme S. Extended tight-binding quantum chemistry methods. Wiley Interdiscip. Rev.: Comput. Mol. Sci. 2021, 11, e1493 10.1002/wcms.1493.

[ref37] Salem L. The Molecular Orbital Theory of Conjugated Systems; W. A. Benjamin, 1972.

[ref38] Hod O.; Rabani E.; Baer R. Magnetoresistance of Nanoscale Molecular Devices. Acc. Chem. Res. 2006, 39, 109–117. 10.1021/ar0401909. 16489730

[ref39] Hod O.; Baer R.; Rabani E. Magnetoresistance of nanoscale molecular devices based on Aharonov–Bohm interferometry. J. Phys.: Condens.Matter 2008, 20, 383201 10.1088/0953-8984/20/38/383201. 21693808

[ref40] Hoffmann R. An Extended Hückel Theory. I. Hydrocarbons. J. Chem. Phys. 1963, 39, 1397–1412. 10.1063/1.1734456.

[ref41] Hoffmann R. Extended Hückel Theory. II. σ Orbitals in the Azines. J. Chem. Phys. 1964, 40, 2745 10.1063/1.1725601.

[ref42] Hoffmann R. Extended Hückel Theory. III. Compounds of Boron and Nitrogen. J. Chem. Phys. 1964, 40, 2474–2480. 10.1063/1.1725550.

[ref43] Hoffmann R. Extended Hückel Theory. IV. Carbonium Ions. J. Chem. Phys. 1964, 40, 2480–2488. 10.1063/1.1725551.

[ref44] Hoffmann R. Extended Hückel theory—v: Cumulenes, polyenes, polyacetylenes and cn. Tetrahedron 1966, 22, 521–538. 10.1016/0040-4020(66)80020-0.

[ref45] Hoffmann R. Extended Hückel theory—vi: Excited states and photochemistry of diazirines and diazomethanes. Tetrahedron 1966, 22, 539–545. 10.1016/0040-4020(66)80021-2.

[ref46] Elstner M.; Porezag D.; Jungnickel G.; Elsner J.; Haugk M.; Frauenheim T.; Suhai S.; Seifert G. Self-consistent-charge density-functional tight-binding method for simulations of complex materials properties. Phys. Rev. B: Condens. Matter Mater. Phys. 1998, 58, 7260–7268. 10.1103/physrevb.58.7260.

[ref47] Frauenheim T.; Seifert G.; Elstner M.; Niehaus T.; Köhler C.; Amkreutz M.; Sternberg M.; Hajnal Z.; Carlo A. D.; Suhai S. Atomistic simulations of complex materials: ground-state and excited-state properties. J. Phys.: Condens.Matter 2002, 14, 3015–3047. 10.1088/0953-8984/14/11/313.

[ref48] Yang Y.; Yu H.; York D.; Cui Q.; Elstner M. Extension of the Self-Consistent-Charge Density-Functional Tight-Binding Method: Third-Order Expansion of the Density Functional Theory Total Energy and Introduction of a Modified Effective Coulomb Interaction. J. Phys. Chem. A 2007, 111, 10861–10873. 10.1021/jp074167r. 17914769

[ref49] Gaus M.; Cui Q.; Elstner M. DFTB3: Extension of the Self-Consistent-Charge Density-Functional Tight-Binding Method (SCC-DFTB). J. Chem. Theory Comput. 2011, 7, 931–948. 10.1021/ct100684s. PMC3509502 23204947

[ref50] Weber W.; Thiel W. Orthogonalization corrections for semiempirical methods. Theor. Chem. Acc. 2000, 103, 495–506. 10.1007/s002149900083.

[ref51] Stewart J. J. P. Optimization of parameters for semiempirical methods V: Modification of NDDO approximations and application to 70 elements. J. Mol. Model. 2007, 13, 1173–1213. 10.1007/s00894-007-0233-4. 17828561 PMC2039871

[ref52] Kromann J. C.; Christensen A. S.; Steinmann C.; Korth M.; Jensen J. H. A third-generation dispersion and third-generation hydrogen bonding corrected PM6 method: PM6-D3H+. PeerJ 2014, 2, e449 10.7717/peerj.449. 25024918 PMC4081274

[ref53] Stewart J. J. P. Optimization of parameters for semiempirical methods VI: more modifications to the NDDO approximations and re-optimization of parameters. J. Mol. Model. 2012, 19, 1–32. 10.1007/s00894-012-1667-x. 23187683 PMC3536963

[ref54] Bannwarth C.; Ehlert S.; Grimme S. GFN2-xTB—An Accurate and Broadly Parametrized Self-Consistent Tight-Binding Quantum Chemical Method with Multipole Electrostatics and Density-Dependent Dispersion Contributions. J. Chem. Theory Comput. 2019, 15, 1652–1671. 10.1021/acs.jctc.8b01176. 30741547

[ref55] Pople J. A. Molecular-Orbital Theory of Diamagnetism. I. An Approximate LCAO Scheme. J. Chem. Phys. 2004, 37, 53–59. 10.1063/1.1732974.

[ref56] Nishimoto K.; Mataga N. Electronic Structure and Spectra of Some Nitrogen Heterocycles. Z. Phys. Chem. 1957, 12, 335–338. 10.1524/zpch.1957.12.5_6.335.

[ref57] Ohno K. Some Remarks on the Pariser-Parr-Pople Method. Theor. Chim. Acta 1964, 2, 219–227. 10.1007/bf00528281.

[ref58] Klopman G. A Semiempirical Treatment of Molecular Structures. II. Molecular Terms and Application to Diatomic Molecules. J. Am. Chem. Soc. 1964, 86, 4550–4557. 10.1021/ja01075a008.

[ref59] Aidas K.; Angeli C.; Bak K. L.; Bakken V.; Bast R.; Boman L.; Christiansen O.; Cimiraglia R.; Coriani S.; Dahle P.; Dalskov E. K.; Ekström U.; Enevoldsen T.; Eriksen J. J.; Ettenhuber P.; Fernández B.; Ferrighi L.; Fliegl H.; Frediani L.; Hald K.; Halkier A.; Hättig C.; Heiberg H.; Helgaker T.; Hennum A. C.; Hettema H.; Hjertenæs E.; Høst S.; Høyvik I.-M.; Iozzi M. F.; Jansík B.; Jensen H. J. A.; Jonsson D.; Jørgensen P.; Kauczor J.; Kirpekar S.; Kjærgaard T.; Klopper W.; Knecht S.; Kobayashi R.; Koch H.; Kongsted J.; Krapp A.; Kristensen K.; Ligabue A.; Lutnæs O. B.; Melo J. I.; Mikkelsen K. V.; Myhre R. H.; Neiss C.; Nielsen C. B.; Norman P.; Olsen J.; Olsen J. M. H.; Osted A.; Packer M. J.; Pawlowski F.; Pedersen T. B.; Provasi P. F.; Reine S.; Rinkevicius Z.; Ruden T. A.; Ruud K.; Rybkin V. V.; Sałek P.; Samson C. C. M.; de Merás A. S.; Saue T.; Sauer S. P. A.; Schimmelpfennig B.; Sneskov K.; Steindal A. H.; Sylvester-Hvid K. O.; Taylor P. R.; Teale A. M.; Tellgren E. I.; Tew D. P.; Thorvaldsen A. J.; Thøgersen L.; Vahtras O.; Watson M. A.; Wilson D. J. D.; Ziolkowski M.; Ågren H.; Ågren H. The Dalton quantum chemistry program system. Wiley Interdiscip. Rev.: Comput. Mol. Sci. 2014, 4, 269–284. 10.1002/wcms.1172. 25309629 PMC4171759

[ref60] Dalton, a molecular electronic structure program, Release v2018.0, 2018. http://daltonprogram.org.

[ref61] Ruud K.; Helgaker T.; Bak K. L.; Jo/rgensen P.; Jensen H. J. A. Hartree–Fock limit magnetizabilities from London orbitals. J. Chem. Phys. 1993, 99, 3847–3859. 10.1063/1.466131.

[ref62] Wolinski K.; Hinton J. F.; Pulay P. Efficient implementation of the gauge-independent atomic orbital method for NMR chemical shift calculations. J. Am. Chem. Soc. 1990, 112, 8251–8260. 10.1021/ja00179a005.

[ref63] Lutnæs O. B.; Teale A. M.; Helgaker T.; Tozer D. J.; Ruud K.; Gauss J. Benchmarking density-functional-theory calculations of rotational g tensors and magnetizabilities using accurate coupled-cluster calculations. J. Chem. Phys. 2009, 131, 144104 10.1063/1.3242081. 19831430

[ref64] Teale A. M.; Lutnæs O. B.; Helgaker T.; Tozer D. J.; Gauss J. Benchmarking density-functional theory calculations of NMR shielding constants and spin–rotation constants using accurate coupled-cluster calculations. J. Chem. Phys. 2013, 138, 024111 10.1063/1.4773016. 23320672

[ref65] Pritchard B. P.; Altarawy D.; Didier B.; Gibson T. D.; Windus T. L. New Basis Set Exchange: An Open, Up-to-Date Resource for the Molecular Sciences Community. J. Chem. Inf. Model. 2019, 59, 4814–4820. 10.1021/acs.jcim.9b00725. 31600445

[ref66] Feller D. The role of databases in support of computational chemistry calculations. J. Comput. Chem. 1996, 17, 1571–1586. 10.1002/(sici)1096-987x(199610)17:13<1571::aid-jcc9>3.0.co;2-p.

[ref67] Schuchardt K. L.; Didier B. T.; Elsethagen T.; Sun L.; Gurumoorthi V.; Chase J.; Li J.; Windus T. L. Basis Set Exchange: A Community Database for Computational Sciences. J. Chem. Inf. Model. 2007, 47, 1045–1052. 10.1021/ci600510j. 17428029

[ref68] Dunning T. H. Gaussian basis sets for use in correlated molecular calculations. I. The atoms boron through neon and hydrogen. J. Chem. Phys. 1989, 90, 1007–1023. 10.1063/1.456153.

[ref69] Kendall R. A.; Dunning T. H.; Harrison R. J.; Harrison R. J. Electron affinities of the first-row atoms revisited. Systematic basis sets and wave functions. J. Chem. Phys. 1992, 96, 6796–6806. 10.1063/1.462569.

[ref70] Woon D. E.; Dunning T. H.; Thom H. Gaussian basis sets for use in correlated molecular calculations. V. Core-valence basis sets for boron through neon. J. Chem. Phys. 1995, 103, 4572–4585. 10.1063/1.470645.

[ref71] Balabanov N. B.; Peterson K. A. Systematically convergent basis sets for transition metals. I. All-electron correlation consistent basis sets for the 3d elements Sc–Zn. J. Chem. Phys. 2005, 123, 064107 10.1063/1.1998907. 16122300

[ref72] Balabanov N. B.; Peterson K. A. Basis set limit electronic excitation energies, ionization potentials, and electron affinities for the 3d transition metal atoms: Coupled cluster and multireference methods. J. Chem. Phys. 2006, 125, 074110 10.1063/1.2335444. 16942325

[ref73] Koput J.; Peterson K. A. Ab Initio Potential Energy Surface and Vibrational-Rotational Energy Levels of X2Σ+ CaOH. J. Phys. Chem. A 2002, 106, 9595–9599. 10.1021/jp026283u.

[ref74] Prascher B. P.; Woon D. E.; Peterson K. A.; Dunning T. H.; Wilson A. K. Gaussian basis sets for use in correlated molecular calculations. VII. Valence, core-valence, and scalar relativistic basis sets for Li, Be, Na, and Mg. Theor. Chem. Acc. 2011, 128, 69–82. 10.1007/s00214-010-0764-0.

[ref75] Wilson A. K.; Woon D. E.; Peterson K. A.; Dunning T. H. Gaussian basis sets for use in correlated molecular calculations. IX. The atoms gallium through krypton. J. Chem. Phys. 1999, 110, 7667–7676. 10.1063/1.478678.

[ref76] Woon D. E.; Dunning T. H. Gaussian basis sets for use in correlated molecular calculations. III. The atoms aluminum through argon. J. Chem. Phys. 1993, 98, 1358–1371. 10.1063/1.464303.

[ref77] Woon D. E.; Dunning T. H. Gaussian basis sets for use in correlated molecular calculations. IV. Calculation of static electrical response properties. J. Chem. Phys. 1994, 100, 2975–2988. 10.1063/1.466439.

[ref78] Bates J. E.; Furche F. Harnessing the meta-generalized gradient approximation for time-dependent density functional theory. J. Chem. Phys. 2012, 137, 164105 10.1063/1.4759080. 23126693

[ref79] Stoychev G. L.; Auer A. A.; Neese F. Automatic Generation of Auxiliary Basis Sets. J. Chem. Theory Comput. 2017, 13, 554–562. 10.1021/acs.jctc.6b01041. 28005364

[ref80] Sauer S. P. A. Molecular Electromagnetism: A Computational Chemistry Approach; Oxford University Press, 2011.

[ref81] Gregor T.; Mauri F.; Car R. A comparison of methods for the calculation of NMR chemical shifts. J. Chem. Phys. 1999, 111, 1815–1822. 10.1063/1.479451.

[ref82] Xiaozeng Y.; Weixiong W. 15N and 17O NMR chemical shift calculations using the MNDO/GIAO method. Magn. Reson. Chem. 1987, 25, 860–863. 10.1002/mrc.1260251006.

[ref83] Patchkovskii S.; Thiel W. NMR chemical shifts in MNDO approximation: Parameters and results for H, C, N, and O. J. Comput. Chem. 1999, 20, 1220–1245. 10.1002/(sici)1096-987x(199909)20:12<1220::aid-jcc3>3.0.co;2-#.

[ref84] Heine T.; Seifert G.; Fowler P. W.; Zerbetto F. A Tight-Binding Treatment for 13C NMR Spectra of Fullerenes. J. Phys. Chem. A 1999, 103, 8738–8746. 10.1021/jp9923062.

[ref85] Vieille L.; Berlu L.; Combourieu B.; Hoggan P. A Quantum Chemistry GIAO molecular site approach of NMR chemical shifts generalized to the whole periodic table. J. Theor. Comput. Chem. 2002, 01, 295–308. 10.1142/s0219633602000245.

[ref86] Wang B.; Brothers E. N.; van der Vaart A.; Merz K. M. Fast semiempirical calculations for nuclear magnetic resonance chemical shifts: A divide-and-conquer approach. J. Chem. Phys. 2004, 120, 11392–11400. 10.1063/1.1752877. 15268173

[ref87] Williams D. E.; Peters M. B.; Wang B.; Merz K. M. MNDO Parameters for the Prediction of 19F NMR Chemical Shifts in Biologically Relevant Compounds. J. Phys. Chem. A 2008, 112, 8829–8838. 10.1021/jp801649f. 18722416

[ref88] Williams D. E.; Peters M. B.; Wang B.; Roitberg A. E.; Merz K. M. AM1 Parameters for the Prediction of 1H and 13C NMR Chemical Shifts in Proteins. J. Phys. Chem. A 2009, 113, 11550–11559. 10.1021/jp9028722. 19799435

[ref89] Teale A. M.; Tozer D. J. Exchange representations in Kohn–Sham NMR shielding calculations. Chem. Phys. Lett. 2004, 383, 109–114. 10.1016/j.cplett.2003.10.138.

[ref90] Hegstrom R. A.; Lipscomb W. N. Paramagnetism in Closed-Shell Molecules. Rev. Mod. Phys. 1968, 40, 354–358. 10.1103/revmodphys.40.354.

[ref91] Fowler P.; Steiner E. Paramagnetic closed-shell molecules: the isoelectronic series CH+, BH and BeH-. Mol. Phys. 1991, 74, 1147–1158. 10.1080/00268979100102871.

[ref92] Sauer S. P. A.; Enevoldsen T.; Oddershede J. Paramagnetism of closed shell diatomic hydrides with six valence electrons. J. Chem. Phys. 1993, 98, 9748–9757. 10.1063/1.464353.

[ref93] Ruud K.; Helgaker T.; Bak K. L.; Jørgensen P.; Olsen J. Accurate magnetizabilities of the isoelectronic series BeH-BH, and CH+. The MCSCF-GIAO approach. Chem. Phys. 1995, 195, 157–169. 10.1016/0301-0104(95)00052-p.

[ref94] Soncini A.; Teale A. M.; Helgaker T.; De Proft F.; Tozer D. J. Maps of current density using density-functional methods. J. Chem. Phys. 2008, 129, 074101 10.1063/1.2969104. 19044754

[ref95] Sundholm D.; Fliegl H.; Berger R. J. Calculations of magnetically induced current densities: theory and applications. Wiley Interdiscip. Rev.: Comput. Mol. Sci. 2016, 6, 639–678. 10.1002/wcms.1270.

[ref96] Jusélius J.; Sundholm D.; Gauss J. Calculation of current densities using gauge-including atomic orbitals. J. Chem. Phys. 2004, 121, 3952–3963. 10.1063/1.1773136. 15332941

[ref97] Krzeszewski M.; Ito H.; Itami K. Infinitene: A Helically Twisted Figure-Eight [12]Circulene Topoisomer. J. Am. Chem. Soc. 2022, 144, 862–871. 10.1021/jacs.1c10807. 34910487

[ref98] Fliegl H.; Taubert S.; Lehtonen O.; Sundholm D. The gauge including magnetically induced current method. Phys. Chem. Chem. Phys. 2011, 13, 20500–20518. 10.1039/c1cp21812c. 21909556

[ref99] Orozco-Ic M.; Valiev R. R.; Sundholm D. Non-intersecting ring currents in [12]infinitene. Phys. Chem. Chem. Phys. 2022, 24, 6404–6409. 10.1039/d2cp00637e. 35262148

[ref100] Ahrens J. P.; Geveci B.; Law C. C. ParaView: An End-User Tool for Large-Data Visualization. The Visualization Handbook; Elsevier, 2005.

[ref101] Ayachit U. The ParaView Guide: A Parallel Visualization Application; Kitware, Inc.: Clifton Park, NY, USA, 2015.

[ref102] Culpitt T.; Peters L. D. M.; Tellgren E. I.; Helgaker T. Ab initio molecular dynamics with screened Lorentz forces. I. Calculation and atomic charge interpretation of Berry curvature. J. Chem. Phys. 2021, 155, 024104 10.1063/5.0055388. 34266267

[ref103] Peters L. D. M.; Culpitt T.; Monzel L.; Tellgren E. I.; Helgaker T. Ab Initio molecular dynamics with screened Lorentz forces. II. Efficient propagators and rovibrational spectra in strong magnetic fields. J. Chem. Phys. 2021, 155, 024105 10.1063/5.0056235. 34266256

[ref104] Culpitt T.; Peters L. D. M.; Tellgren E. I.; Helgaker T. Analytic calculation of the Berry curvature and diagonal Born–Oppenheimer correction for molecular systems in uniform magnetic fields. J. Chem. Phys. 2022, 156, 044121 10.1063/5.0079304. 35105065

[ref105] Monzel L.; Pausch A.; Peters L. D. M.; Tellgren E. I.; Helgaker T.; Klopper W. Molecular dynamics of linear molecules in strong magnetic fields. J. Chem. Phys. 2022, 157, 054106 10.1063/5.0097800. 35933207

[ref106] Peters L. D. M.; Culpitt T.; Tellgren E. I.; Helgaker T. Magnetic-translational sum rule and approximate models of the molecular Berry curvature. J. Chem. Phys. 2022, 157, 134108 10.1063/5.0112943. 36208997

[ref107] Qiao Z.; Welborn M.; Anandkumar A.; Manby F. R.; Miller T. F. OrbNet: Deep learning for quantum chemistry using symmetry-adapted atomic-orbital features. J. Chem. Phys. 2020, 153, 124111 10.1063/5.0021955. 33003742

[ref108] Qiao Z.; Ding F.; Welborn M.; Bygrave P. J.; Smith D. G. A.; Anandkumar A.; Manby F. R.; Miller T. F. Multi-task learning for electronic structure to predict and explore molecular potential energy surfaces. 2020, arXiv:2011.02680.

