# Supplementary material for: Semiempirical Methods for Molecular Systems in Strong Magnetic Fields
Source: J Chem Theory Comput. 2023 Sep 6;19(18):6226–41. doi: 10.1021/acs.jctc.3c00671 (PMC10536997; doi:10.1021/acs.jctc.3c00671)
Supplement: Supplementary file 1 — ct3c00671_si_001.pdf [file ct3c00671_si_001.pdf]

Supplementary Information for: Semi-Empirical Methods for Molecular Systems in Strong Magnetic Fields

Chi Y. Cheng[1] and Andrew M. Wibowo-Teale[1,2]

[1]School of Chemistry, University of Nottingham, University Park, Nottingham, NG7 2RD, UK
[2]Hylleraas Centre for Quantum Molecular Sciences, Department of Chemistry, University of Oslo, P.O. Box 1033 Blindern, N-0315 Oslo, Norway

Relative Timings

In this section, we determine the computational costs for the GFN1-xTB-M methods relative to the GFN1-xTB method using QUEST. We also include relative timings with the GFN1-xTB method implemented using xTB [1] and the HF/STO-6G and HF/3-21G methods with QUEST for comparison purposes. Calculations were carried out in the absence of a magnetic field, GFN1-xTB calculations used AOs and GFN1-xTB-M0, GFN1-xTB-M1, HF/STO-6G and HF/3-21G calculations were forced to use LAOs. Comparisons are made by calculating single-point energies of cyclooctatetraene (COT) carried out with a single-core on the Intel Xeon Gold 6138 @ 2.00 GHz CPU, results are shown in Table 1. We can see an increased computational cost of 2.8 times for the GFN1-xTB-M0 and GFN1-xTB-M1 methods. The increased cost will be due to the additional kinetic energy integrals evaluated and the complex number arithmetic required in the GFN1-xTB-M methods. Even with the increased costs, the GFN1-xTB-M methods are still considerably more computationally inexpensive than the HF methods.

Program	Method	Relative Timings
xTB	GFN1-xTB	0.4
QUEST	GFN1-xTB	1.0
QUEST	GFN1-xTB-M0	2.8
QUEST	GFN1-xTB-M1	2.8
QUEST	HF/STO-6G	54000
QUEST	HF/3-21G	3900

Table 1: Relative timings for the single-point energy calculations of COT in the absence of a magnetic field using xTB or QUEST with a single-core on the Intel Xeon Gold 6138 @ 2.00 GHz CPU. GFN1-xTB calculations used AOs while GFN1-xTB-M0, GFN1-xTB-M1, HF/STO-6G and HF/3-21G calculations used LAOs.

Magnetic Property Calculations

Full results of the GFN1-xTB-M and HF magnetizabilities and NMR shielding constants calculations.

Molecule	GFN1-xTB-M0	GFN1-xTB-M1	HF/STO-6G	HF/3-21G	CCSD(T)†
HF	-133.0	-147.2	-139.4	-156.4	-176.4
CO	-94.0	-197.6	-124.1	-192.8	-209.5
N_2	-69.9	-217.7	-128.8	-178.5	-205.2
H_2O	-168.2	-201.7	-192.9	-211.6	-235.1
HCN	-170.0	-329.2	-221.7	-276.1	-271.8
HOF	-200.3	-198.3	-217.5	-242.0	-235.4
O_3	129.2	33.1	1619.5	1248.6	121.5
NH_3	-189.9	-257.6	-253.5	-271.7	-290.3
H_2CO	-119.4	-214.9	-89.0	-124.6	-127.4
CH_4	-249.0	-342.4	-305.5	-324.0	-316.9
C_2H_4	-244.4	-404.8	-326.3	-361.3	-345.6
AlF	-327.7	-316.0	-262.1	-401.0	-394.5
CH_3F	-279.7	-327.9	-338.8	-326.4	-315.7
C_3H_4	-361.8	-513.7	-489.4	-506.5	-478.9
FCCH	-346.8	-497.8	-418.5	-471.9	-441.6
FCN	-267.2	-393.0	-324.7	-385.2	-370.0
H_2S	-325.4	-448.2	-391.5	-474.3	-455.1
HCP	-263.0	-511.2	-389.4	-518.1	-492.8
HFCO	-227.6	-296.4	-232.6	-306.8	-307.2
H_2C_2O	-328.3	-490.3	-379.1	-455.9	-423.9
LiF	-228.9	-248.6	-175.5	-195.2	-195.5
LiH	-135.2	-152.4	-127.5	-138.9	-127.2
N_2O	-261.0	-384.2	-295.8	-349.8	-339.1
OCS	-403.2	-586.7	-505.2	-624.6	-584.1
OF_2	-230.6	-201.1	-252.6	-277.9	-247.1
H_4C_2O	-431.3	-492.4	-559.3	-563.1	-535.2
PN	25.1	-215.1	-104.7	-267.2	-308.2
SO_2	-84.8	-257.2	326.5	-199.8	-314.3

Table 2: GFN1-xTB-M0, GFN1-xTB-M1, HF/STO-6G, HF/3-21G and CCSD(T) calculated isotropic magnetizabilities in $10^{-30}\mathrm{JT}^{-2}$. †CCSD(T) values extrapolated to the basis set limit which were obtained from Ref. [2].

Molecule	Nucleus	GFN1-xTB-M0	GFN1-xTB-M1	HF/STO-6G	HF/3-21G	CCSD(T)†
HF	H	36.42	32.50	34.83	30.64	28.83
	F	61.51	-27.14	415.13	422.30	420.31
CO	O	-240.75	-333.07	-0.62	-66.38	-55.05
	C	-303.01	-208.37	12.98	8.39	2.24
N_2	N	-422.92	-307.68	-16.46	-90.93	-60.43
H_2O	O	33.35	-2.38	364.91	341.11	338.01
	H	30.25	31.23	33.77	32.96	30.65
HCN	N	-328.27	-239.66	48.47	-13.90	-14.11
	C	-142.61	-82.33	132.75	99.54	84.58
	H	25.80	34.31	29.3	29.85	29.01
HOF	O	-150.49	-294.18	154.64	-15.45	-68.92
	H	23.84	23.96	27.94	22.89	19.57
	F	-106.85	-467.92	181.69	252.07	192.21
O_3	O_{mid}	-621.19	-870.74	-3046.19	-3957.61	-763.70
	O_{term}	-873.38	-1223.94	-3882.98	-4037.27	-1221.62
NH_3	N	-34.96	-18.36	310.06	273.93	270.66
	H	29.51	34.24	33.40	33.91	31.44
H_2CO	H	21.36	26.84	22.73	22.50	21.91
	O	-388.06	-578.69	-438.66	-474.81	-378.61
	C	-230.03	-162.00	87.25	32.41	1.53
CH_4	C	-22.93	-5.76	236.26	211.82	198.93
	H	30.76	36.16	32.99	33.01	31.30
C_2H_4	C	-186.39	-121.65	118.56	92.90	69.71
	H	23.93	30.69	27.58	27.43	26.05
AlF	F	46.64	107.33	347.24	227.94	211.85
CH_3F	F	28.76	-209.36	502.37	503.03	482.88
	C	-75.28	-50.07	192.53	156.15	122.15
	H	29.28	33.70	31.62	29.41	27.35
C_3H_4	C_3	-50.29	-27.42	237.29	213.18	192.10
	C_1	-178.55	-113.40	127.67	104.32	83.69
	H_1	23.43	30.12	26.92	25.95	24.37
	H_3	30.60	34.24	33.06	32.33	30.64
FCCH	F	-18.92	-237.43	451.16	436.43	423.55
	C_H	-72.29	-38.03	205.29	187.01	179.86
	C_F	-97.89	-56.86	167.52	134.53	100.06
	H	30.04	36.21	31.74	31.44	30.49
FCN	F	-50.16	-280.74	399.25	381.29	374.10
	N	-229.09	-164.31	122.36	101.28	117.89
	C	-107.52	-58.09	140.21	108.05	82.24
H_2S	H	26.84	30.28	34.38	32.82	30.45
HCP	C	-170.56	-112.03	79.92	41.33	37.55
	H	24.45	33.32	31.04	30.46	29.56

Table 3: GFN1-xTB-M0, GFN1-xTB-M1, HF/STO-6G, HF/3-21G and CCSD(T) calculated isotropic NMR shielding constants in ppm. †CCSD(T) values extrapolated to the basis set limit which were obtained from Ref. [3].

Molecule	Nucleus	GFN1-xTB-M0	GFN1-xTB-M1	HF/STO-6G	HF/3-21G	CCSD(T)†
HFCO	H	24.67	28.76	25.23	24.91	23.86
	F	-104.04	-400.53	295.91	225.93	165.27
	C	-170.13	-115.26	110.86	67.61	39.63
	O	-250.92	-385.71	-170.44	-135.59	-94.33
H_2C_2O	H	27.75	33.39	31.39	31.29	29.19
	C_H	-67.23	-37.27	207.15	201.29	193.32
	C_O	-216.58	-141.34	68.62	31.08	-6.34
	O	-196.91	-296.79	-15.37	-20.82	-5.92
LiF	F	70.54	-6.83	296.70	341.14	382.48
LiH	H	30.54	29.91	26.08	26.16	26.58
N_2O	N_{end}	-213.17	-157.31	100.56	60.59	106.45
	N_{cent}	-217.74	-166.11	87.57	-2.29	12.56
	O	-83.01	-151.38	138.75	137.95	199.02
OCS	O	-137.55	-215.02	119.43	64.59	96.76
	C	-169.53	-108.56	72.89	39.31	30.18
OF_2	O	-290.22	-500.84	-17.30	-258.06	-447.09
	F	-136.12	-525.88	142.65	70.57	-23.95
H_4C_2O	O	-46.25	-191.31	378.71	384.40	363.23
	C	-65.60	-36.11	209.96	181.70	153.20
	H	29.57	33.69	32.50	31.32	29.14
PN	N	-690.02	-549.23	-459.70	-523.69	-343.97
SO_2	O	-344.66	-516.23	-1609.98	-704.56	-242.68

Table 3: *Continued*

Benzene Current Densities

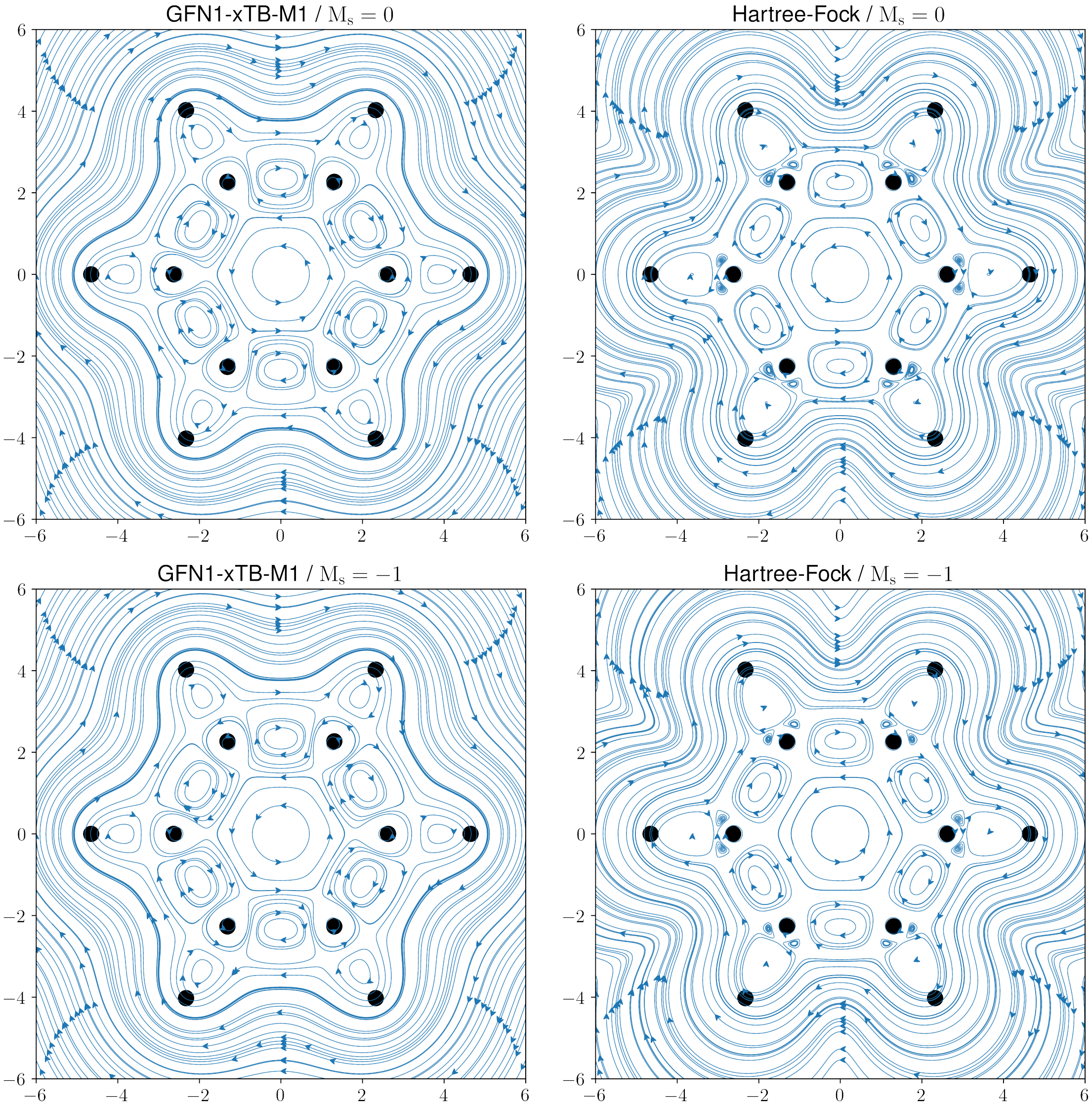

Figure 1: Streamline plots of the GFN1-xTB-M1 and HF/*u*-aug-cc-pVDZ current densities of benzene with the closed-shell $\mathrm{M_s} = 0$ and open-shell $\mathrm{M_s} = -1$ electronic configurations under a magnetic field oriented along the z-axis with $\mathcal{B}_z = 0.1\mathrm{B}_0$. The benzene molecule lies in the xy-plane and streamlines are plotted across the molecular plane.

References

[1] *Semiempirical Extended Tight-Binding Program Package*. https://github.com/grimme-lab/xtb.

[2] Ola B. Lutnæs et al. "Benchmarking density-functional-theory calculations of rotational g tensors and magnetizabilities using accurate coupled-cluster calculations". In: *J. Chem. Phys.* 131.14 (2009), p. 144104.

[3] Andrew M. Teale et al. "Benchmarking density-functional theory calculations of NMR shielding constants and spin–rotation constants using accurate coupled-cluster calculations". In: *J. Chem. Phys.* 138.2 (2013), p. 024111.
